# Prevalence, incidence, and risk factors for herpes zoster in systemic lupus erythematosus: a systematic review and meta-analysis

**DOI:** 10.3389/fimmu.2025.1544218

**Published:** 2025-08-01

**Authors:** Hong-Fei Wang, Yan Gao, Zheng Lin, Shan Liu, Yi Cao, Qiu-Shuang Li

**Affiliations:** ^1^ The First Affiliated Hospital of Zhejiang Chinese Medical University (Zhejiang Provincial Hospital of Chinese Medicine), Hangzhou, China; ^2^ First School of Clinical Medicine, Zhejiang Chinese Medicine University, Hangzhou, China; ^3^ Department of Rheumatology and Immunology, The First Affiliated Hospital of Zhejiang Chinese Medical University (Zhejiang Provincial Hospital of Chinese Medicine), Hangzhou, China; ^4^ Research and Development Department, The First Affiliated Hospital of Zhejiang Chinese Medical University (Zhejiang Provincial Hospital of Chinese Medicine), Hangzhou, China

**Keywords:** systemic lupus erythematosus, herpes zoster, incidence, prevalence, risk factor

## Abstract

**Background:**

Patients with systemic lupus erythematosus (SLE) are particularly vulnerable to infections, with herpes zoster (HZ) being the most common opportunistic infection. This meta-analysis aimed to systematically review the available literature on the prevalence, incidence, and risk factors of HZ in SLE patients.

**Methods:**

A comprehensive search through Embase, PubMed, Web of Science, and Cochrane Library was conducted for studies published up to November 1, 2024. Both observational studies (including cohort, case-control, and cross-sectional) and randomized controlled trials (RCTs) were included, with study types selected according to the specific objectives. Funnel plots and Egger’s test were employed to assess publication bias. Hazard ratios (HRs) and odds ratios (ORs) were converted to relative risks (RRs), and pooled estimates were calculated using a fixed-effect or random-effects model.

**Results:**

A total of 51 studies with 246, 822 SLE patients were included in this meta-analysis. The pooled prevalence and incidence of SLE-HZ were 12.3% (95%CI 10.5-14.1) and 22.0 cases per 1000 person-years (95%CI 17.4-27.9). Glucocorticoids use (RRs=2.83, 95%CI 2.10-3.81), cyclophosphamide use (RRs=2.52, 95%CI 1.60-3.98), mycophenolate mofetil use (RRs=3.00, 95%CI 1.07-8.40), azathioprine use (RRs=1.40, 95%CI 1.18-1.67), anifrolumab use (RRs=2.59, 95%CI 1.52-4.41), having lymphopenia (RRs=2.31, 95%CI 1.54-3.46), and the presence of comorbid conditions such as renal involvement (RRs= 1.80, 95%CI 1.34-2.42) were identified to increase the risk of HZ in SLE patients.

**Conclusion:**

The existing evidence highlights the both high prevalence and incidence of HZ in SLE patients. By identifying risk factors associated with the development of HZ in SLE patients, optimization of management strategies and treatment choices can be achieved. Concurrently, physicians could be better equipped to choose patients who would most likely gain from the HZ vaccine.

**Systematic review registration:**

https://www.crd.york.ac.uk/PROSPERO/view/CRD42024331310, identifier CRD42024331310.

## Introduction

Herpes zoster (HZ) arises from the reactivation of the latent varicella-zoster virus (VZV). It is particularly prevalent and prone to dissemination in geriatric and immunocompromised individuals, potentially posing a life-threatening risk (1). Among these populations, systemic lupus erythematosus (SLE) patients are particularly susceptible to VZV reactivation (2, 3). The incidence of herpes zoster infection (HZI) among SLE patients ranges from 2.54 to 91.4 cases per 1000 patient-years (PYs) (2, 4–6), with a risk that is 2-8 times greater than other rheumatic diseases and 5-16 times higher than in the general population (7–9). SLE patients also face an elevated risk for postherpetic neuralgia (PHN), which is 2.3 times higher than the general population (10).

Although fatalities related to HZI in SLE patients are rarely reported, hospitalizations related to HZ have notably increased in recent years (11, 12). The burden of HZ and PHN on healthcare systems is substantial, significantly impacting both patient well-being and quality of life. Recent reviews suggested a focus on preventing specific infections in SLE, particularly VZV, due to the potential increased risks associated with new therapies (13). Hence, identifying risk factors for HZ in the SLE population is imperative for early detection and intervention.

Although successive studies have reported that the prevalence and incidence of HZ, as well as factors such as high-dose glucocorticoids (GCs) therapy, immunosuppressants, comorbidities (e.g., renal insufficiency), and autoantibodies, may contribute to the risk of HZI (14–17), the results are inconsistent (18–21). This inconsistency may be attributed to differences in sample size, conducted region, and patient characteristics. Thus, our systematic review aims to assess the prevalence, incidence, and associated factors of HZ in studies involving SLE patients. Furthermore, we will attempt to aggregate data related to HZ events as much as possible.

## Methods

This systematic review followed the Preferred Reporting Items for Systematic Reviews and Meta-Analyses (PRISMA) guidelines (22), which help ensure transparency and reproducibility in systematic reviews. The protocol was pre-registered in PROSPERO (CRD 42024331310).

### Inclusion and exclusion criteria

We included studies published in English with full-text availability that reported outcomes related to the prevalence, incidence, or risk factors of HZ among SLE patients. Different study designs were included according to the specific objective of each analysis. Cohort, case-control, and cross-sectional studies were used to estimate prevalence. Only cohort studies were included to assess incidence. For evaluating risk factors, we included cohort and case-control studies. In addition, when assessing the efficacy and safety of biological agents in patients with SLE, some randomized controlled trials (RCTs) have reported the incidence of HZ as an adverse outcome. Accordingly, these RCTs were included in our analysis to estimate the relative risk of HZ associated with biologic therapy.

SLE patients were required to meet the American College of Rheumatology (ACR) criteria (23, 24) or the International Classification of Diseases (ICD-9-CM code 710.0; ICD-10-CM code M32.0-M32.1, M32.9). HZ cases were identified based on physician-reported ICD codes (ICD-9-CM code 053; ICD-10-CM code B02). Those diagnosed by typical vesicular eruption developing in a dermatomal distribution were also included, as recommended by the 2016 European consensus guidelines (25). Only patients with a history of HZ following the diagnosis of SLE were considered. Studies that did not meet the inclusion criteria were excluded, along with letters, reviews, commentaries, conference abstracts, and case reports.

### Literature search

We searched Embase, PubMed, Web of Science, and Cochrane Library from their inception to November 1, 2024. The search strategy incorporated medical subject headings (MeSH) and free-text keywords, including ‘herpes zoster’, ‘ systemic lupus erythematosus’, ‘prevalence’, ‘incidence’, ‘risk factor’, and their associated variations. The full search strategy is documented in **Supplementary Table S1**. A thorough search was conducted by screening the bibliographies of every relevant study manually. According to the predefined inclusion and exclusion criteria, two independent reviewers screened the literature based on titles and abstracts, followed by a full-text review. Discrepancies were resolved by consulting a third reviewer.

### Data extraction and quality assessment

Two researchers independently extracted data from the included studies, with subsequent cross-checking to ensure accuracy. Data encompassed study characteristics: the first author, publication year, study design, country, study period; population characteristics: total number of subjects, age, gender; outcome definitions: diagnostic methods for SLE and HZ, number of HZ patients, reported prevalence and incidence rates (IRs) with 95% confidence intervals (CIs), and risk factors associated with the outcomes. When reported, data on recurrence, hospitalization, dermatologic involvement sites, and complications were extracted. For risk factors, priority was given to extracting adjusted confounders. No additional information was sought from the original authors.

The quality of each cohort or case-control study was assessed by the same reviewers using the Newcastle-Ottawa Quality Assessment Scale (NOS) (26), while cross-sectional study quality was evaluated using the Agency for Healthcare Research and Quality (AHRQ) guidelines (27). RCTs were assessed by the Cochrane risk of bias tool. Any disagreements were resolved by QS-L.

### Statistical analysis

For prevalence estimates, the numerator was the number of HZ after SLE onset, while the denominator was the total number of SLE patients. Incidence rate was calculated by dividing the number of new cases by the total person time, with results presented per 1000 patient-years. Recurrence was defined as the subsequent episodes after the initial occurrence in HZ patients. Risk factors reported in at least two studies were analyzed using pooled RRs for dichotomous variables and weighted mean differences (WMDs) for continuous variables such as SLE disease duration. Given the low incidence of HZ (approximately 10%), OR was treated as the reasonable approximation of RR when pooling the risk factors (28). HR was directly considered as RR, consistent with the approach used in the previous study (29, 30). Additionally, relative risk was calculated to evaluate the risk of receiving biologic drugs (31). When data could not be combined due to substantial clinical or methodological heterogeneity or insufficient studies, the results were reported qualitatively. Heterogeneity was deemed significant when *I* ^2 ^ exceeded 50% or when *P* value in the Q test was below  0.1 (32), prompting the application of a random effects model (33). Subgroup analyses were performed to explore potential sources of heterogeneity based on geographical region (Asia vs. North America vs. South America vs. Europe vs. Africa), publication year (before 2014 vs. 2014-2024), sample size (<100 vs. 100-1000 vs. >1000), and diagnostic modality of SLE and HZ [classic clinical manifestations vs. unclear (identify patient from ICD codes)]. Furthermore, we conducted additional meta-regressions to investigate potential sources of heterogeneity. The independent variables included were consistent with those applied in the subgroup analysis. Sensitivity analysis was performed by sequentially omitting individual studies. Egger’s regression and funnel plots were used to evaluate publication bias in meta-analyses with 10 or more papers (34). Subsequent sensitivity analysis using the trim-and-fill procedure was carried out to identify potential “missing studies” and investigate their impact on the pooled effect estimate, in cases where significant publication bias was detected (35, 36). The interaction test for subgroups were undertaken by Review Manager V.5.4, while the other data analyses were conducted using Stata version 18.0 (StataCorp, LLC, College Station, TX, authorized by The First Affiliated Hospital of Zhejiang Chinese Medical University).

## Results

### Study selection

**Figure 1:**
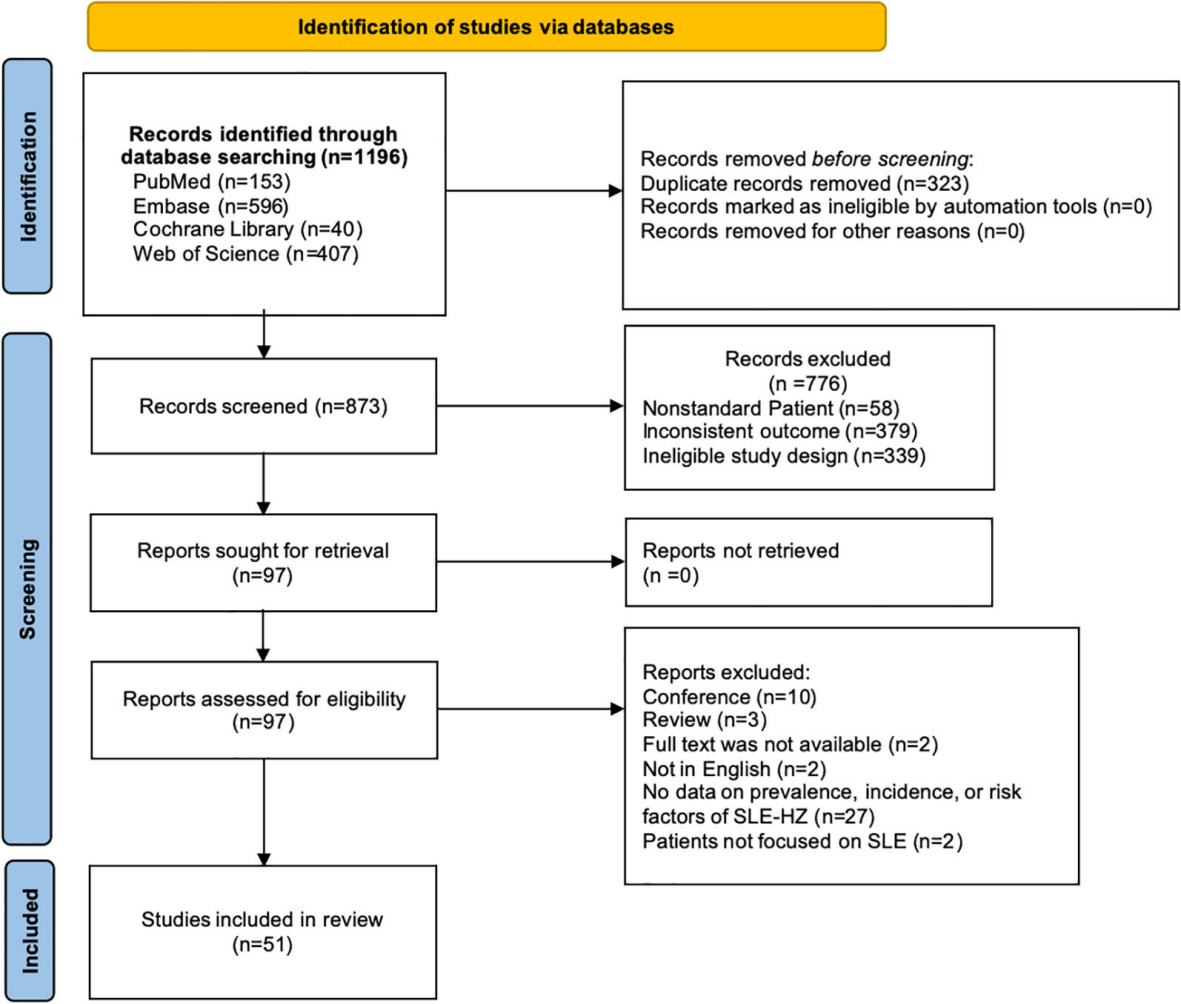
PRISMA flow chart.

Our literature found a total of 1,196 records. After eliminating duplicates (n=323) and studies that did not meet the eligibility criteria (n=776), 51 eligible studies were ultimately included. Of these, 14 studies addressed both prevalence/incidence and risk factors, 28 studies focused solely on prevalence or incidence, and 9 studies were dedicated exclusively to risk factors (**Figure 1**). All studies included were published between 1978 to 2024 and consisted of 26 cohort studies, 10 case-control studies, 5 cross-sectional studies, and 10 RCTs. Sample sizes varied from 29 to 21,255 SLE patients, of which seventeen studies enrolled >1000 patients. Females constituted the majority of the enrolled population. The detailed characteristics are displayed in **Table 1**. All studies were classified as moderate to high quality. (**Supplementary Table S2**)

**Table 1:** Characteristics of the included studies.

Study	Country	Study design	Study period	N	Reported outcomes (prevalence/incidence rate)	Inclusion criteria		SLE patients’ characteristics		Reported significant risk factors
						SLE	HZ	Mean age, years	Gender (female%)	
Borba, 2010 (5)	Brazil	Cohort	1999.1-2006.6	51/1145	4.45%; 6.4/1000 PYs	ACR-1997	Typical clinical findings	39 ±13.7	90.2	NA
Bruce, 2021 (37)	Multicenter	RCT	2017.3-2017.10	4/36	11%*	ACR-1997	NA	44.9±10.6	89.0	8
Chakravarty, 2013 (8)	USA	Cohort	2001.1-2009.12	409/1485	16.2 (12.4-21.2)/1000 PYs	Questionnaire	Typical clinical findings	48.3 ±13	93.5	1, 4
Chen, 2011 (39)	China	Cohort	1996-2006	NA/10337	37.7 (35.5-40.0)/1000 PYs	ICD-9-CM and ACR-1982/1997	ICD-9-CM	34.8 ± 14.3	89.8	NA
Chen, 2014 (40)	USA	Cohort	2005.1-2009.12	3540/144137	15.19 (14.69-15.69) /1000 PYs	ICD-9-CM	ICD-9-CM	46.9±13.2	87.8	NA
Chen, 2016 (41)	China	Case-control	2005.6-2015.6	86/3815	NA	ACR-1997	Typical clinical findings	35.0 ± 13.2	83.0	NA
Chen, 2017 (14)	China	Case-control	2009.1-2013.1	46/1265	3.60%	ACR-1997	Typical clinical findings	35.0±14.0	95.7	1,11,15,18
Costa-Reis, 2013 (42)	USA	Cohort	2007-2009	8/120	13/1000 PYs	ACR-1997	Documentation of the microorganism and/or typical clinical findings	NA	78.0	NA
Da Silva, 2020 (43)	Brazil	Cohort	1990.1-2014.12	27/92	29.3%; 11.3/1000 PYs	Medical records	Typical clinical findings	10.64 ± 3.11	88.9	NA
Feldman, 2015 (44)	USA	Cohort	2000.1-2006.12	160/5068	NA	ICD-9-CM	ICD-9-CM	38.8±12.48	93.5	NA
Ferreira, 2016 (45)	Brazil	Cohort	2012.11-2014.10	120/852	14%	ACR-1997	Typical clinical findings	11±3.98	89.0	1,3,11,19
Frodlund, 2024 (15)	Sweden	Cohort	2005.7-2019.12	319/5309	6%	ICD-10-CM	ICD-10-CM	49.9±18.2	84.6	2
Furie, 2017 (46)	Multicenter	RCT	2012.1-2014.1	17/305	NA	ACR-1997	NA	40.8±11.6	93.4	8
Furie, 2019 (47)	Multicenter	RCT	2015.6-2017.6	18/457	NA	ACR-1982/1997	NA	NA	92.3	8
Garnier, 2018 (48)	France	Cohort	2011.1-2015.12	3/29	34.5 (11.8-96.6)/1000 PYs	ACR-1997	Medical chart reviews	37.3 ± 15.0	79.3	NA
Gormezano, 2015 (49)	Brazil	Cross-sectional	NA	108/2192	NA	ACR-1997	Typical clinical findings	NA	NA	NA
Hata, 2011 (4)	Japan	Cohort	2001.19-2007.12	38/1077	53.7/1000 PYs	ICD-10-CM	ICD-10-CM	48.23±19.29	76.6	NA
Hsu, 2019 (50)	China	Cohort	2000.1-2013.12	2580/15961	28.9 (27.8-30.0)/1000 PYs	ACR-1997	ICD-9-CM	37.2 ± 17.0	87.3	NA
Hu, 2013 (21)	China	Case-control	1999.12-2008.12	65/170	NA	ACR-1982	Typical clinical findings	33.92 ±12.68	90.8	3,11,12,13,15,16
Hu, 2016 (17)	China	Case-control	2000-2009	1555/8410	NA	ICD-9-CM	ICD-9-CM	36.44±16.87	88.9	1,3,4,5,6,7
Ishikawa, 1999 (51)	Japan	Cross-sectional	NA	27/58	46.60%	NA	NA	40.2 ± 13.4	NA	NA
Kahl, 1994 (16)	USA	Cross-sectional	NA	47/348	13.5%; 16.0/1000 PYs	Medical records	Typical clinical findings	NA	NA	NA
Kalunian, 2023 (52)	Multicenter	RCT	2016.6-2020.3	30/369	8.9%*; 3.4/1000 PYs	ACR-1982/1997	NA	43.4 ± 12.0	92.1	8
Kang, 2005 (6)	Korea	Cohort	1991.1-2000.12	42/303	32.5/1000 PYs	ACR	Typical clinical findings	34.1 ± 11.6	84.8	14
Khalifa, 2007 (53)	Tunisia	Cross-sectional	1990.1-2004.12	3/75	4%	ACR	Documentation of the microorganism and/or clinical findings	31.4 ± 13.5	85.3	NA
Kwan, 2022 (54)	Canada	Cohort	2016.5-2018.11	82/422	30.5%; 14.0 (11.5-17.7)/1000 PYs	ACR-1982	Typical clinical findings	29.3±12.0	95.1	1,11
Lee, 2006 (18)	China	Cohort	1988.1-2004.7	15/49	30.6%; 58.7/1000 PYs	ACR-1982	Typical clinical findings	10.87 ± 3.61	91.8	
Manzi, 1995 (55)	USA	Case-control	1979-1989	48/321	15%	ACR-1982	Typical clinical findings	NA	NA	NA
Merrill, 2010 (56)	Multicenter	RCT	NA	19/257	NA	ACR	NA	40.2±11.4	91.0	9
Mok, 2023 (2)	China	Cohort	2019.3-2019.8	161/573	28.1%; 2.54/1000 PYs	Medical records	Typical clinical findings	35.2 ± 14.2	93.7	NA
Morand, 2020 (57)	Multicenter	RCT	2015.7-2018.9	15/362	NA	ACR-1997	NA	43.1±12.0	93.4	8
Moutsopoulos, 1978 (58)	USA	Cohort	NA	13/83	21.70%	Clinical diagnosis	Typical clinical findings	25±12.75	80.7	NA
Murray, 2016 (11)	USA	Cross-sectional	2000-2011	NA	NA	ICD-9-CM	ICD-9-CM	51 ± 17	89.0	NA
Nagasawa, 1990 (59)	Japan	Cohort	NA	36/92	43.50%	ACR-1982	Typical clinical findings	36.8±12.25	96.7	NA
Nishimaki, 1999 (60)	Japan	Case-control	1975-1996	22/132	17%	ACR-1982/1997	Typical clinical findings	27.9 ± 11.7	NA	NA
Park, 2004 (61)	Korea	Cohort	1990.1-2000.12	42/303	13.86%; 32.5/1000 PYs	ACR-1997	Typical clinical findings	27.42 ± 6.63	100.0	NA
Pope, 2004 (62)	Canada	Case-control	NA	6/61	NA	ACR-1982	Typical clinical findings	49±15.62	93.0	NA
Rodziewicz, 2023 (63)	UK	Cohort	2010.7-2021.2	8/929	NA	ACR-1997	NA	NA	89.9	9
Rovin, 2012 (64)	Multicenter	RCT	2006.1-2008.1	20/144	15.1%*	ACR-1997	NA	31.8±9.6	87.5	9
Ryu, 2021 (7)	Korea	Cohort	2009.1-2013.10	515/21255	10.2 (9.32-11.08)/1000 PYs	KCD-6-CM	KCD-6 code	NA	NA	NA
Sayeeda, 2010 (65)	Saudi	Case-control	1982-2006	32/624	5.10%	ACR-1982	Typical clinical findings	31.4 ± 11.4	93.8	NA
Sheikh, 2021 (66)	Multicenter	RCT	2012.11-2017.7	48/4003	NA	ACR-1982/1997	NA	40.4 ±12.75	92.0	10
Stohl, 2017 (67)	Multicenter	RCT	2011.11-2015.2	31/836	3.2%*	ACR-1997	NA	38.1 ± 12.10	94. 4	NA
Strom, 1994 (68)	USA	Case-control	1985-1987	18/195	9.20%	ACR-1982	Personal interviews and chart reviews	NA	89.2	NA
Teh, 2018 (69)	Malaysia	Cohort	2011.1-2015.12	14/125	NA	ACR-1997	Typical clinical findings	33.4 ± 14.2	89.6	NA
Wu, 2011 (70)	China	Cohort	1999.12-2008.6	35/98	35.70%	ACR-1982	Typical clinical findings	12.85±2.43	85.4	1,3
Yang, 2018 (19)	China	Cohort	NA	128/1850	6.92%; 29.3/1000 PYs	ICD-9-CM	ICD-9-CM	47.76 ±16.57	83.7	1, 17
Yu, 2022 (71)	China	Cohort	NA	126/1042	12.10%	ICD-9-CM	ICD-9-CM	35.0±16.2	89.5	NA
Yun, 2016 (8)	USA	Cohort	2007-2010	NA/8320	20.0/1000 PYs	ICD-9-CM	ICD-9-CM	NA	NA	NA
Zamora, 2020 (20)	Filipino	Case-control	2009.1-2014.12	65/626	NA	Medical records	Typical clinical findings	36.75±1.35	93.8	1,3,4,7,15,18
Zhang, 2018 (72)	Multicenter	RCT	2011.5-2015.9	41/705	6.2%*	ACR-1997	NA	32.3 ±9.65	92.9	10

1. Steroid dose, 2. Immunosuppressants, 3. CTX, 4. MMF, 5. AZA, 6. MTX, 7.HCQ, 8. Anifrolumab, 9. Rituximab, 10. Belimumab, 11. Lymphopenia, 12. Anti-Ro, 13. Anti-RNP, 14. Anti-Sm, 15. Renal involvement, 16. Neuropsychiatric manifestations, 17. Chronic liver disease, 18. Active lupus, 19. Disease duration <1 year, 20. Other major infections.

HZ, herpes zoster; SLE, systemic lupus erythematosus; ACR, American College of Rheumatology; ICD, International Classification of Diseases; PYs, patient-years; N, number of patients (SLE patients with HZ/ Total SLE patients)

*Prevalence in the medical-intervention group.

### Prevalence of HZ in SLE

**Figure 2:**
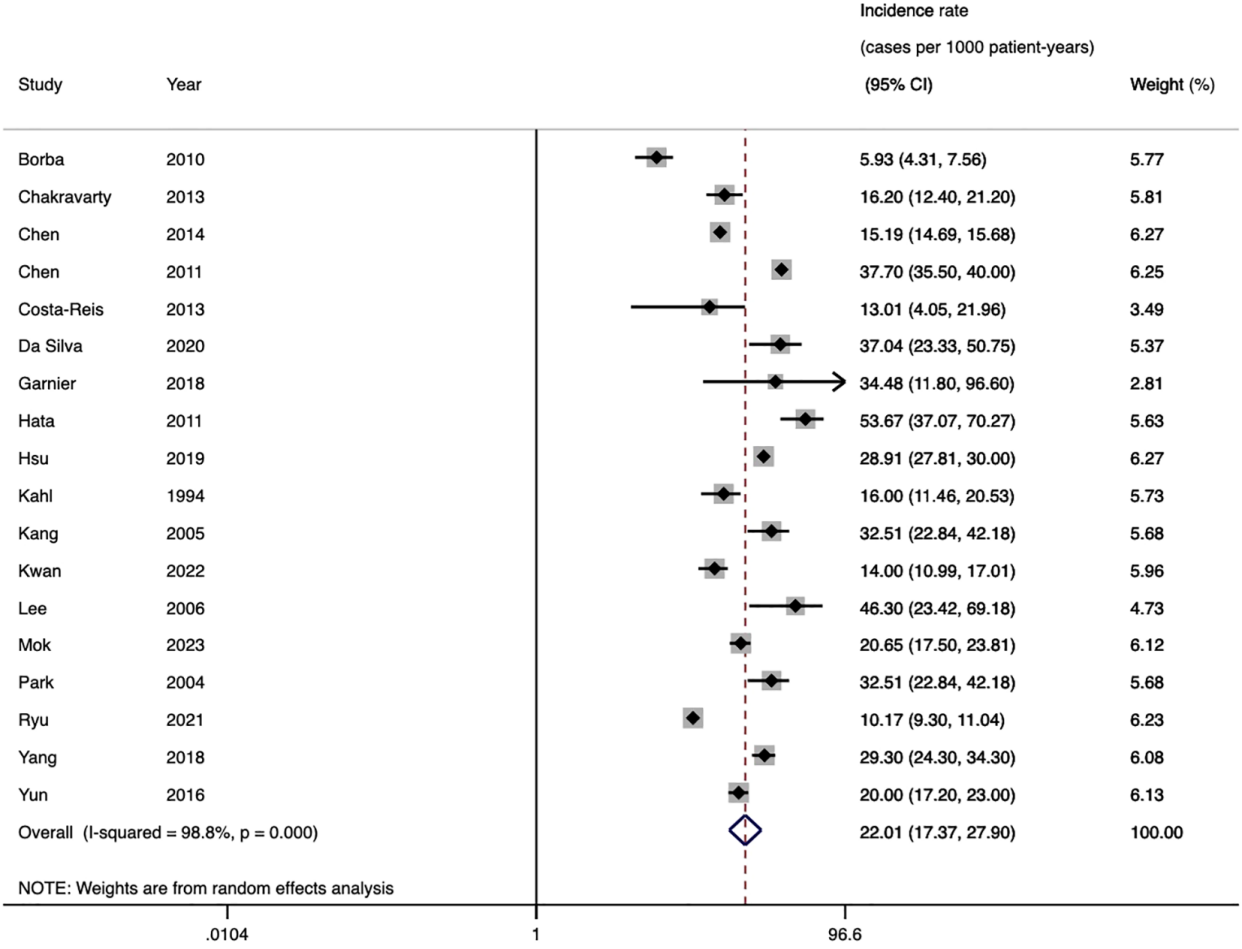
Forest plot of SLE-HZ prevalence. SLE, systemic lupus erythematosus; HZ, herpes zoster.

Thirty studies (including cohort, case-control, and cross-sectional designs) reported the prevalence of HZ, with a total of 3,949 patients with HZ among 56,783 SLE patients. The pooled prevalence was 12.3% (95%CI 10.5-14.1, *I* ^2^ = 98.7%), indicating substantial heterogeneity (**Figure 2**). Subgroup analysis results revealed the highest prevalence was observed in Asia (14.8%, 95%CI 11.7-17.9), followed by North America (12.2%, 95%CI 6.0-18.3), and South America (10.4%, 95%CI 6.2-14.6). Only one study each was from Europe and Africa. Studies with less than 100 participants had a higher prevalence (25.8%) than those with 100–1000 participants (13.8%) and more than 1000 participants (6.3%). The prevalence in the past decade was 10.5% (95%CI 8.2-12.8), which was lower than the prevalence rate in the previous decade (16.1%, 95%CI 12.2-19.9). Furthermore, prevalence based on the ACR-1982 (16.4%) was higher than ACR-1997 (7.3%) and ICD codes (8.2%). Diagnoses based on typical clinical findings showed a higher prevalence of HZ than ICD codes (13.5 vs 8.2%) (**Table 2**). The results of the meta-regression analyses revealed that sample size influenced the pooled HZ prevalence among patients with SLE significantly (**Supplementary Table S3**).

**Table 2:** Subgroup analysis for the prevalence and incidence of SLE-HZ.

Subgroups	N	Effect size (95% CI)	*I* ^2^(%)
Prevalence (%)			
**Overall**	31^*^	12.3 (10.5-14.1)	98.7
Region			
Asia	17	14.8 (11.7-17.9)	99.2
North America	7	12.2 (6.0-18.3)	96.0
South America	5	10.4 (6.2-14.6)	96.1
Europe	1	6.0 (5.4-6.7)	NA
Africa	1	4.0 (0.8-11.2)	NA
Sample size			
<100	8	25.8 (14.5-37.1)	93.1
100-1000	13	13.8 (10.5-17.2)	92.6
>1000	10	6.3 (3.8-8.8)	99.4
Publication year			
Before 2014	15	16.1 (12.2-19.9)	93.4
2014-2024	16	10.5 (8.2-12.8)	99.2
Diagnostic criteria of SLE^†^			
ACR-1982	11	16.4 (11.4-21.3)	93.1
ACR-1997	8	7.3 (5.1-9.5)	95.7
Medical records	2	19.3 (0.8-37.9)	93.3
Unclear (identify patient from ICD codes)	6	8.2 (4.0-12.3)	99.7
Diagnostic criteria of HZ			
Classic clinical manifestations	23	13.5 (11.1-15.8)	96.7
Unclear (identify patient from ICD codes)	6	8.2 (4.0-12.3)	99.7
Incidence (per 1000 person-years)			
**Overall**	18	22.0 (17.4-27.9)	98.8
Region			
Asia	9	26.0 (19.0-35.6)	98.2
North America	6	18.7 (12.1-29.1)	97.1
South America	2	14.8 (2.5-88.7)	99.2
Europe	1	34.5 (11.8-96.6)	NA
Publication year			
Before 2014	9	23.5 (14.9-36.9)	96.5
2014-2024	9	20.5 (15.2-27.8)	99.1
Diagnostic criteria of SLE			
ACR-1982	4	23.2 (14.1-38.3)	96.1
ACR-1997	5	18.7 (8.9-39.2)	97.4
Unclear (identify patient from ICD codes)	6	23.6 (14.9-37.2)	99.5
Diagnostic criteria of HZ			
Classic clinical manifestations	11	20.5 (14.8-28.6)	92.0
Unclear (identify patient from ICD codes)	7	24.2 (16.8-35.0)	99.6

N, number of data points; HZ, herpes zoster; SLE, systemic lupus erythematosus; ACR, American College of Rheumatology; ICD, International Classification of Diseases; NA, not available.

^*^Gormezano et al. (2015) reported prevalence separately for two distinct cohorts: childhood SLE and adult SLE, resulting in a total of 31 data points from 30 studies.

**^†^** Several studies lacked data on diagnostic criteria of SLE.

Publication bias was evident in the asymmetrical funnel plot, with a significant Egger’s test result (*P*<0.001) (**Supplementary Figure S1**). To address this, we employed the trim-and-fill method to explore the impact of “missing studies” on the pooled prevalence. After including the two “missing” studies, the new pooled estimate was less variable at 11.3 (95% CI 9.5-13.1), indicating the robustness of the previous results despite potential publication bias. Sensitivity analysis indicated no significant changes in the overall estimates after omitting any study (**Supplementary Figure S2**).

### Incidence rate of HZ in SLE

**Figure 3:**
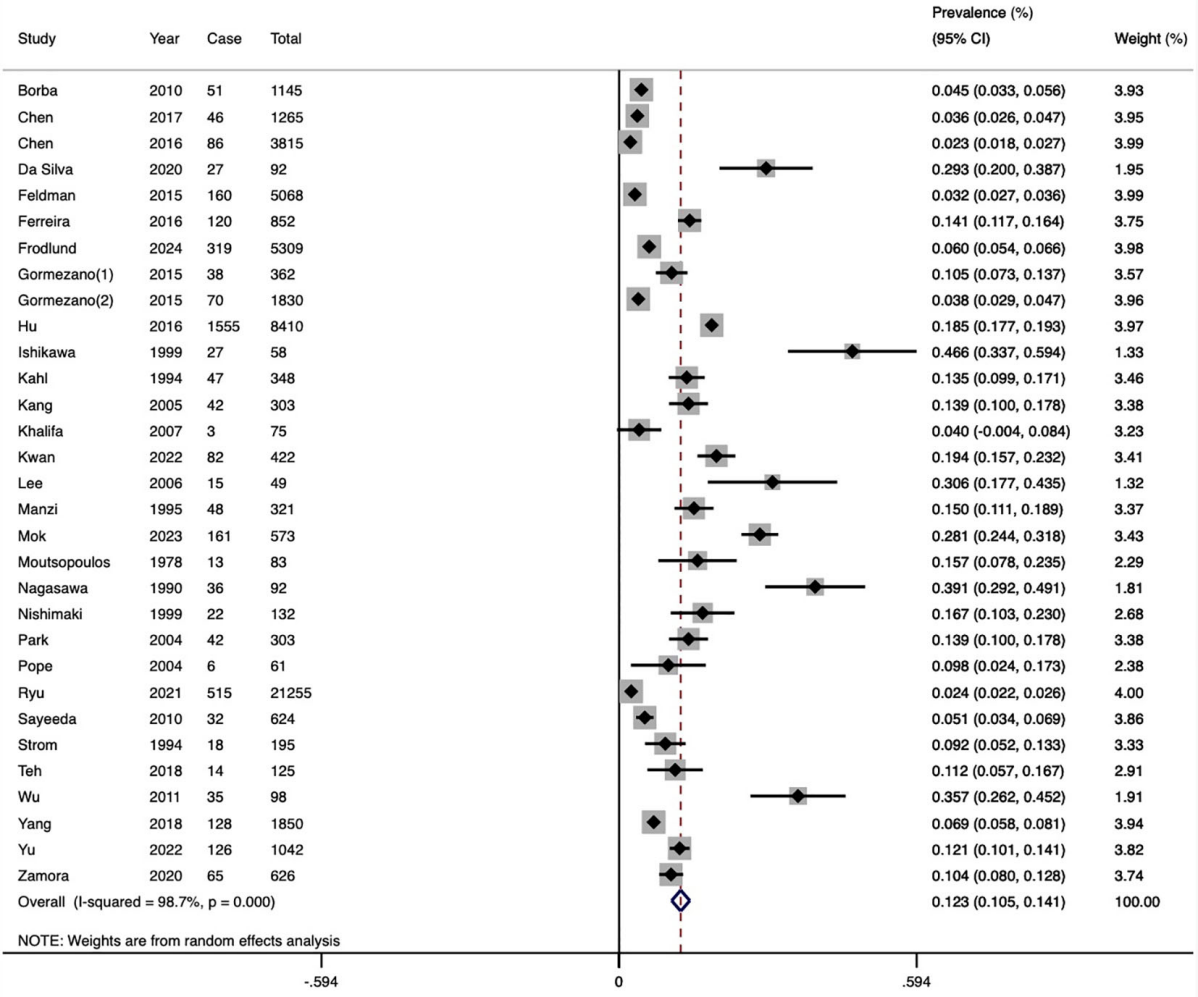
Forest plot of SLE-HZ incidence. SLE, systemic lupus erythematosus; HZ, herpes zoster.

The pooled incidence of HZ across 18 cohort studies was 22.0 cases per 1,000 patient-years (95%CI 17.4-27.9, *I* ^2^ = 98.8%) (**Figure 3**). Stratified by region, the highest incidence was observed in Asia (26.0/1000 PYs), followed by North America (18.7/1000 PYs) and South America (14.8/1000 PYs). The studies published ten years ago reported higher incidence (23.5/1000 PYs) than the recent ten years (20.5/1000 PYs). Diagnoses based on typical clinical findings showed a lower incidence of HZ than ICD codes (20.5/1000 PYs vs 24.2/1000 PYs). Subgroup analysis based on diagnostic criteria of SLE revealed consistent results between ICD codes and ACR-1982 groups (23.2/1000 PYs vs 23.6/1000 PYs). Diagnoses based on ACR-1997 showed a lower incidence (18.7/1000 PYs) (**Table 2**). None of the above factors were considered to be significant contributors to heterogeneity in incidence subgroup analyses and meta-regression analyses (**Supplementary Table S3**).

Sensitivity analysis revealed no significant changes in the incidence estimates after omitting any individual study (**Supplementary Figure S3**). Funnel plots and Egger’s meta-regression tests (t=0.17, *P*=0.869) indicated no significant publication bias (**Supplementary Figure S4**).

### Risk factors associated with HZ in SLE

Risk factors were categorized into three groups: demographics, which include gender and age at SLE diagnosis; clinical features including duration between the onset of SLE and HZ, lupus disease activity, complications of HZ (e.g., renal involvement, neuropsychiatric manifestations); laboratory data, such as autoantibody, leucopenia, lymphopenia; therapeutic variables include GCs, mycophenolate mofetil (MMF), cyclophosphamide (CTX), azathioprine (AZA), cyclosporine A (CsA), methotrexate (MTX), antimalarial [chloroquine and hydroxychloroquine (HCQ)], and biological drugs (belimumab, anifrolumab, and rituximab).

Among the demographic factors, our pooled effects did not find any significant association between older age at SLE diagnosis (RRs=1.01, 95%CI 1.00-1.02) (15, 38, 54) and female (RRs=0.98, 95%CI 0.72-1.31) (15, 38, 54) with the development of HZ. Patients with renal involvement were more likely to be HZ (RRs= 1.80, 95%CI 1.34-2.42) (14, 18–21, 70), while no significant association was found with neuropsychiatric manifestations (RRs=1.29, 95%CI 0.55-3.02) (18, 19, 21, 70). Longer SLE disease duration also showed no correlation with HZ occurrence (WMD=-0.41, 95%CI -2.09 - 1.27) (18, 20, 21, 45, 54). The included studies assessed lupus disease activity using the SLE Disease Activity Index (SLEDAI) or the Safety of Estrogens in Lupus Erythematosus National Assessment-SLEDAI score. Patients were defined as active SLE if the SLEDAI score was ≥6 or SELENA-SLEDAI > 3 (73, 74). In our meta-analysis, the combined results of two studies showed no statistically significant association between active lupus and SLE-HZ (RRs=1.04, 95%CI 0.14-7.83) (14, 20). Regarding clinical factors, three studies found lymphopenia (lymphocyte count < 1,500/mm^3^) was inclined to be HZ (RRs=2.31, 95%CI 1.54-3.46) (14, 21, 45). Based on the medication used, we found using GCs was associated with a higher risk of HZI (RRs= 2.83, 95%CI 2.10-3.81) (14, 15, 17–21, 38, 45, 54, 70). Specifically, long-term oral prednisone (RRs=3.60, 95%CI 3.03-4.29) (17, 21, 45, 70) posed a greater risk than intravenous methylprednisolone therapy (RRs=2.15, 95%CI 1.71-2.70) (17, 18, 45, 70). Additionally, the use of immunosuppressants is linked to an increased occurrence of HZ (RRs=1.60, 95%CI 1.31-1.96) (15, 17–21, 38, 45, 54, 70). Pooled effects indicated that receiving CTX (RRs=2.52, 95%CI 1.60-3.98) (17, 18, 20, 21, 45, 65, 70), MMF (RRs=3.00, 95%CI 1.07-8.40) (17, 18, 20, 38), AZA (RRs=1.40, 95%CI 1.18-1.67) (17, 18, 20) therapies were all associated with a higher risk of HZI. No association was found with antimalarial drugs (RRs=0.99, 95%CI 0.64-1.55) (15, 17, 20, 38, 54). Patients receiving anifrolumab tends to develop HZ (RRs=2.59, 95%CI 1.52-4.41) (37, 46, 47, 52, 57), while we don’t find the significant result from rituximab and belimumab (RRs=1.67, 95%CI 0.88-3.18; RRs=0.75, 95%CI 0.52-1.09) (**Table 3**).

**Table 3:** Meta-analysis of risk factors for SLE-HZ.

Outcomes	N	ES (95%CI)	*P*-value	*I* ^2^ (%)
Demographic				
Older age at SLE diagnosis, years	3	1.01 (1.00-1.02)	0.054	0.0
Female (vs male)	3	0.98 (0.72-1.31)	0.865	36.0
Clinical				
Longer SLE disease duration	5	-0.41 (-2.09-1.27) ^*^	0.631	83.9
Active lupus	2	1.04 (0.14-7.83)	0.969	90.3
Having renal involvement	6	**1.80 (1.34-2.42)**	< 0.01	39.9
Having neuropsychiatrie manifestations	4	1.29 (0.55-3.02)	0.558	59.7
Laboratory				
Having lymphopenia	3	**2.31 (1.54-3.46)**	< 0.01	0.0
Therapeutic				
Glucocorticoid use	16	**2.83 (2.10-3.81)**	< 0.01	69.9
Methylprednisolone pulse therapy	5	**2.15 (1.71-2.70)**	< 0.01	1.8
Oral prednisolone	5	**3.60 (3.03-4.29)**	< 0.01	46.8
Immunosuppressive agents use	23	**1.60 (1.31-1.96)**	< 0.01	62.7
Mycophenolate mofetil use	4	**3.00 (1.07-8.40)**	< 0.01	57.0
Cyclophosphamide use	8	**2.52 (1.60-3.98)**	< 0.01	67.7
Azathioprine use	3	**1.40 (1.18-1.67)**	< 0.01	0.0
Antimalarial use	5	0.99 (0.64-1.55)	0.975	87.1
Anifrolumab use	5	**2.59 (1.52-4.41)**	< 0.01	15.5
Rituximab use	3	1.67 (0.88-3.18)	0.116	0.0
Belimumab use	3	0.75 (0.52-1.09)	0.128	36.8

N, number of data points; ES, effect size; CI, confidence interval.

^*^Risk factors in at least two included studies were weighed using the pooled relative risks (RRs) for dichotomous variables and weighted mean differences (WMDs) for continuous variables (SLE disease duration), respectively.

^#^Bold values indicate statistically significant results with *P* < 0.01.

Funnel plot (**Supplementary Figure S5**) and Egger’s tests indicated no significant publication bias in GCs usage (*P*=0.591). Visual inspection of the funnel plot (**Supplementary Figure S6**) suggested some asymmetry in immunosuppressants usage, with two studies falling outside the funnel boundaries. This variability may be due to that this analysis included all types immunosuppressants, leading to heterogeneity among studies. However, Egger’s test indicated no significant publication bias (*P*=0.135).

### Other clinical manifestations of HZ in SLE

Seventeen studies reported the recurrence rate of HZ, with 131 out of 878 patients experiencing multiple episodes. The pooled analysis of recurrence rate was 13.2% (95%CI 9.1%-17.2%, **Supplementary Figure S7**). Five studies reported the hospitalization of HZ in SLE patients, and the pooled effects was 23.5% (95%CI 7.2-39.8, **Supplementary Figure S8**). Pooled data from six studies showed that approximately 8.3% of patients developed disseminated HZ (**Supplementary Figure S9**). A summary of eleven studies indicated that 13.1% of patients with HZI developed post-herpetic neuralgia (**Supplementary Figure S10**). In the classification of dermatomal distribution reported in 6 studies, the thoracic nerve (54.8%) was the most common nerve involved by herpes zoster, followed by lumbar (14.5%), cranial (10.1%), cervical (9.6%), and sacral nerves (9.3%).

## Discussion

Our review systematically explored the existing evidence regarding the prevalence, incidence, and risk factors for HZ in patients with SLE. Due to immune system deficiencies, SLE patients experience a considerable reduction in VZV-specific CD4 T cells, which elevates their risk of developing HZ (61). The high overall prevalence and incidence of HZ observed in our study (12.3% and 22.0 cases per 1000 person-years, respectively) further indicate that HZ is a prevalent infection among SLE patients. The occurrence of HZ varies across geographical groups. An elevated rate has been noted in Asian populations, particularly in Japan, which is consistent with prior study outcomes (75). The underlying reasons remain unclear, although it has been suggested that genetic predisposition, environmental factors, socioeconomic factors, and medical conditions that differ across regions may contribute (76). Notably, the data from Asia were relatively derived from studies with smaller sample sizes, which may result in less robust estimates of the incidence and prevalence of SLE-HZ. Further research is warranted to discover why SLE patients in Asia, particularly in East Asia, are at a greater risk for HZ. Our results indicate that the burden of HZ among SLE patients have decreased in the past decade, likely reflecting advancements in SLE management strategies, improved therapies, and increased vaccine accessibility (77–80). Both subgroup analyses and meta-regression analyses evaluating the prevalence indicated that sample size may influence the pooled results. As studies employed a small sample size commonly reported a higher prevalence, the longitudinal studies involving larger cohorts of SLE are necessary. In contrast, the incidence data showed no significant between-subgroup heterogeneity across the same variables. This may be explained by the smaller number of included studies and the greater consistency in study design, thereby reducing methodological variation. Moreover, incidence reflects new-onset HZ events, which are less likely to be influenced by historical or cumulative factors than prevalence.

Furthermore, the HZ recurrence rate (13.2%) and PHN (13.1%) reported in our study were both greater than the general population (81). As one of the most significant complications of HZ, PHN often persists for years and is difficult to treat (82). These complications contribute to increased hospitalization rates and impose a substantial economic burden on patients. Thus, it is of great importance to identify and evaluate the factors that may predispose SLE patients to the development of HZ.

A total of 14 factors were summarized and explored, among which lymphopenia, renal involvement, GCs use, immunosuppressive agents use (CTX, MMF, AZA), and anifrolumab use were predisposing factors. Female sex is generally recognized as a risk factor for HZ (3), but our study did not identify a significant association. This discrepancy may be attributed to the predominance of females in the SLE cohort, which may mitigate the impact of gender on HZ risk. As we know, HZ is predominantly observed in elderly individuals and less frequent in those under 18 in both the general population and patients with other immunodeficiency conditions (8). Interestingly, current research indicated that the occurrence of HZ may be more common in the younger SLE group (18-30 years) (8, 39). Compared to healthy children, the occurrence of HZ in pediatric SLE patients could be up to 40-fold higher (18). This may result from fulminant renal injury and greater disease severity at a younger age, requiring higher doses and longer durations of GCs and immunosuppressive drugs, which in turn leads to latent virus reactivation (71, 83). Previous studies have often suggested an association between HZ and active lupus, with a greater tendency to develop dissemination (65). The inflammatory environment during disease flares may alter immune cell function, impacting VZV-specific B cell activation and subsequent antibody production (84). However, several studies have found that HZ occurs during periods of SLE remission more often (16, 20, 45). Additionally, no direct correlation between SLE disease activity and HZI was identified in our analysis. The observed heterogeneity may be attributed to confounding clinical factors such as differences in treatment regimens. As noted in one study (54), SLEDAI scores were not associated with HZ events in multivariable analysis when GCs dosing was excluded. This supports the hypothesis that the observed association between SLEDAI and HZ may be driven by the inclusion of glucocorticoid dosing (a factor independently associated with HZ risk). Laboratory markers associated with disease activity, including elevated anti-dsDNA antibodies, increased ESR, elevated CRP, and decreased complement levels (C3, C4), were also not found to be correlated with an increased risk of HZ.

Beyond the inherent immune dysfunction, medications play a critical role in increasing risk of HZ among SLE patients. Patients with SLE often require GCs and other immunosuppressive drugs to control disease activity. While these medications effectively manage disease progression by suppressing the hyperactive humoral immunity, they simultaneously impair cellular immunity, increasing susceptibility to infections (17). Our findings further corroborate this risk. Notably, the use of high-dose GCs (≥30 mg prednisone or ≥1 mg/kg/d, or equivalent dose) was significantly attached to an increased risk of infection. Moreover, we found that intravenous methylprednisolone had a lower risk than oral prednisone, further underscoring the critical role of GC usage patterns in infection risk. Long-term exposure to oral prednisone during maintenance therapy results in cumulative immunosuppressive effects, whereas methylprednisolone pulse therapy is typically used for short-term pulse therapy, reducing the cumulative side effects. This finding supports that the use of repeated methylprednisolone pulses combined with tapered oral prednisone can improve the complete remission rate in lupus nephritis (LN) and reduce corticosteroid adverse effects (85, 86). Our results also showed that SLE patients receiving other immunosuppressive therapies, including CTX, MMF, AZA, were much more likely to develop HZ. Using two or more immunosuppressive medications may result in cumulative inhibitory effects particularly, thereby incrementally elevating the risk of HZ (17). In addition, the dose of therapeutic drug use often correlates to lupus activity. Increased dosages and more intensive use of immunosuppressive agents are often necessary to manage active LN and prevent further renal damage, which explains why complications such as renal involvement, neuropsychiatrie manifestations, and other organ dysfunctions may indirectly be risk factors. As the standard of care for SLE, prior research has found that HCQ offers a pleiotropic protective effect against infection (20). However, our pooled analysis also did not identify a significant impact of antimalarial drugs on HZ occurrence. Furthermore, influenced by disease activity and medication treatment, specific immune alterations such as lymphopenia are linked to an increased risk of HZ (87). Anti-Ro and anti-RNP antibodies have been identified as risk factors for HZ, possibly because these autoantibodies may induce lymphocyte apoptosis or impair lymphocyte function (21). Consequently, the presence of these autoantibodies may disrupt cellular immunity, thereby accounting for the greater risk of HZ in these patients.

Emerging target-specific biological drugs, such as anifrolumab, belimumab, rituximab, sifalimumab, are effective treatment options for SLE (88). Nevertheless, some of these therapies are associated with an increased risk of infections. Our findings indicate that using anifrolumab further increases the incidence of clinically relevant HZ episodes. Given the close connection between type I interferon (IFN) signaling and antiviral immunity, it is not surprising that anifrolumab, therapeutic targeting of the type I IFN system, is linked to an increased risk of HZ (89). In addition, among patients receiving anifrolumab, a high risk of HZ may also be associated with the presence of active LN, likely due to the secondary immunodeficiency and underlying kidney disease (78). While anifrolumab may improve long-term outcomes in SLE patients with or without LN, it has raised concerns about a heightened risk of severe viral infections, including but not limited to HZ but potentially including COVID-19. Besides, HZ may also reactivate as a result of rituximab’s suppression of B cells (63). In contrast, belimumab has demonstrated efficacy in SLE treatment, reducing the need for steroids while not significantly raising infections risk (90). Likewise, ustekinumab has also not been found to promote opportunistic infections during its use (91). Additionally, recent studies have proposed that non-immunosuppressive therapies, such as sodium-glucose cotransporter 2 inhibitors (SGLT2i), may also provide potential therapeutic value for patients with LN and other SLE comorbidities without significantly increasing the risk of HZI (92, 93). Thus, for patients with moderate to severe SLE, particularly those with active manifestations such as LN, it is advisable to optimize disease management by utilizing biologic agents (e.g., belimumab) or other therapies to lessen reliance on GCs and lower the long-term infection risk (63). Regardless, treatment strategies should take into account the biologic agents type and the patient’s comorbid conditions. Further research is warranted to better understand how to balance the therapeutic efficacy of biologics with their potential adverse effects.

The emergence of novel therapeutics for SLE, such as interferon inhibitors, has heightened the focus on preventing HZ. Although guidelines advocate the use of the live-attenuated vaccine for the prevention of herpes zoster in healthy adults (>60 years), data on the application of vaccines in immunocompromised hosts are scarce, as their administration is generally contraindicated in these patients. Large retrospective studies have demonstrated that, regardless of medication status, the HZ vaccine effectively reduces the incidence of HZ over a 2-year follow-up period, providing approximately 5 years of protection for autoimmune disease patients (80). Recent studies indicate that SLE patients exhibit good tolerance to the newer recombinant subunit vaccine (Shingrix), with no reports of severe adverse events or disease flares (94). Therefore, the HZ vaccine can be considered in patients with SLE before intensive immunosuppressive therapy. However, it is best given when SLE is in a stable phase and minimal immunosuppression is required vaccination (95, 96). This aligns with the 2019 EULAR guidelines on vaccination in patients with autoimmune inflammatory rheumatic diseases, including SLE (80). Besides, studies have suggested that adults with underlying comorbidities associated with an elevated risk of HZ may be candidates for earlier vaccination, based on a cost-effectiveness evaluation (3). Vaccination against VZV may also help mitigate the specific risk associated with targeted biologic therapies, such as anifrolumab, in this vulnerable population. Future studies should incorporate vaccination status to better evaluate its protective effects and identify optimal immunization strategies in this vulnerable population.

### Strengths and limitations

Our study possesses several advantages. Firstly, this systematic review and meta-analysis provides a comprehensive analysis of the prevalence, incidence, recurrence, and potential risk factors for HZ in SLE patients. The higher incidence rate relative to prevalence implies that HZ might have a shorter disease course and a higher cure rate. Secondly, the majority of the data included were multivariable-adjusted effect estimates, effectively controlling the influence of confounding factors (such as age, sex, etc.).

However, some shortcomings should be noted. Although our results did not indicate significant publication bias, some eligible studies may not have been fully accessible, and negative findings might have remained unpublished. As a result, certain data related to prevalence, incidence, and risk factors may have been missed. Among the studies we included, only one was conducted in Africa and one in Europe, indicating an underrepresentation that may limit the generalizability of the findings. To address this concern, we conducted an additional analysis excluding these two studies. The pooled estimate remained consistent at 13% (95% CI 0.11-0.15, **Supplementary Figure S11**), indicating that the limited geographic diversity had minimal impact on the overall results. Nevertheless, caution is still needed when extrapolating our findings to populations in underrepresented regions. Further research from these areas such as Africa and Europe is warranted to improve the global applicability of the evidence. Despite controlling for potential confounding factors and excluding outlier studies, significant heterogeneity remains in the data. This may be due to inherent limitations of meta-analysis, such as variability in study design and populations (97, 98). Besides, some risk factors, such as disease activity, were reported in only a few studies, which may lead to biased effect estimates. Additionally, abnormal laboratory markers [e.g., anti-IFN-α autoantibodies (99), anti-Ro and anti-RNP antibodies (21)] may contribute to the prediction of HZ. However, due to sufficient clinical data, further exploration of these factors is not feasible. Consequently, more well-designed, large-scale, prospective studies employing standardized data collection and reporting procedures are needed to better elucidate the role of these factors in the development of HZ in SLE patients.

## Conclusion

The existing evidence demonstrates that the prevalence and incidence of HZ are significantly higher among SLE patients compared to the general population, especially in those with active LN. Medication regimens and relevant laboratory markers offer important predictive information for assessing the risk of HZ. Therefore, future efforts are required to raise awareness of infections among SLE patients and develop preventive strategies, including vaccination and appropriate drug management.

## Data Availability

The original contributions presented in the study are included in the article/**Supplementary Material**. Further inquiries can be directed to the corresponding authors.
