# Supplementary material for: Prevalence, incidence, and risk factors for herpes zoster in systemic lupus erythematosus: a systematic review and meta-analysis
Source: Front Immunol. 2025 Aug 1;16:1544218. doi: 10.3389/fimmu.2025.1544218 (PMC12354570; doi:10.3389/fimmu.2025.1544218)
Supplement: Supplementary file 1 [file Table1.docx]

**Table S1.** Search strategy used for the electronic databases.

| Database | Search strategy | Outcome |
| --- | --- | --- |
| PubMed | ((((("Lupus Erythematosus, Systemic"[Mesh]) OR (Systemic Lupus Erythematosus[Title/Abstract])) OR (Lupus Erythematosus Disseminatus[Title/Abstract])) OR (Libman Sacks Disease[Title/Abstract])) AND (((("Epidemiology"[Mesh]) OR (Epidemiology[Title/Abstract])) OR (risk[Title/Abstract])) OR ((((((("Risk Factors"[Mesh]) OR ("Incidence"[Mesh])) OR ("Prevalence"[Mesh])) OR (Morbidity[Title/Abstract])) OR (Incidence[Title/Abstract])) OR (Prevalence[Title/Abstract])) OR (Risk Factors[Title/Abstract])))) AND ((((("Herpes Zoster"[Mesh]) OR (Shingles[Title/Abstract])) OR (Zona[Title/Abstract])) OR (Zoster[Title/Abstract])) OR (Herpes Zoster[Title/Abstract])) | 153 |
| Embase | #14. #9 AND #12 AND #13  #13. #1 OR #10  #12. #2 OR #11  #11. 'herpes zoster':ti,ab,kw OR shingles:ti,ab,kw OR zona:ti,ab,kw OR zoster:ti,ab,kw  #10. 'systemic lupus erythematosus':ti,ab,kw OR 'lupus erythematosus disseminatus':ti,ab,kw OR 'libman sacks disease':ti,ab,kw  #9. #3 OR #4 OR #5 OR #6 OR #7 OR #8  #8. 'incidence':ti,ab,kw OR 'prevalence':ti,ab,kw OR 'morbidity':ti,ab,kw OR 'risk factor':ti,ab,kw OR 'epidemiology':ti,ab,kw  #7. 'morbidity'/exp  #6. 'epidemiology'/exp  #5. 'risk factor'/exp  #4. 'prevalence'/exp  #3. 'incidence'/exp  #2. 'herpes zoster'/exp  #1. 'systemic lupus erythematosus'/exp | 596 |
| WOS | TS=(Lupus Erythematosus, Systemic OR Systemic Lupus Erythematosus OR SLE OR Lupus Erythematosus Disseminatus OR Libman Sacks Disease) AND TS=(Herpes Zoster OR Shingles OR Zona OR Zoster OR Herpes Zoster OR HZ) AND TS=(epidemiology OR risk OR Risk Factors OR Incidence OR Prevalence OR Morbidity) | 407 |
| Cochrane Library | #1 (prevalence):ti,ab,kw  #2 (morbidity):ti,ab,kw  #3 MeSH descriptor: [Prevalence] explode all trees  #4 MeSH descriptor: [Incidence] explode all trees  #5 (incidence):ti,ab,kw  #6 MeSH descriptor: [Epidemiology] explode all trees  #7 (epidemiology):ti,ab,kw  #8 MeSH descriptor: [Risk Factors] explode all trees  #9 (risk factor):ti,ab,kw  #10 (predictor):ti,ab,kw  #11 ("systemic lupus erythematosus"):ti,ab,kw (Word variations have been searched)  #12 MeSH descriptor: [Lupus Erythematosus, Systemic] explode all trees  #13 (Lupus Erythematosus, Systemic):ti,ab,kw  #14 (SLE):ti,ab,kw  #15 ("lupus erythematosus disseminatus"):ti,ab,kw  #16 ("Libman Sacks disease"):ti,ab,kw  #17 #11 OR #12 OR #13 OR #14 OR #15 OR #16  #18 MeSH descriptor: [Herpes Zoster] explode all trees  #19 (herpes zoster):ti,ab,kw  #20 (Shingles):ti,ab,kw  #21 (Zona):ti,ab,kw  #22 (HZ):ti,ab,kw  #23 #18 OR #19 OR #20 OR #21 OR #22  #24 #1 OR #2 OR #3 OR #4 OR #5 OR #6 OR #7 OR #8 OR #9 OR #10  #25 #17 AND #23 AND #24 | 40 |

**Table S2.** The quality of included studies in meta-analysis.

**(A) Study quality of case-control studies**

| **Author** | **Is the case definition adequate?** | **Representativeness of the Cases** | **Selection of Controls** | **Definition of Controls** | **Comparability of Cases and Controls on the Basis of the Design or Analysis** | **Ascertain-**  **ment**  **of exposure** | **Same method of ascertainment for cases and controls** | **Non-**  **Response rate** | **Total scores** |
| --- | --- | --- | --- | --- | --- | --- | --- | --- | --- |
| Chen, 2017 | ★ | ★ | ★ | ★ | ★★ | ★ | ★ | ☆ | 8 |
| Chen, 2016 | ★ | ★ | ★ | ★ | ★★ | ★ | ★ | ☆ | 8 |
| Hu, 2013 | ★ | ★ | ★ | ★ | ★★ | ★ | ★ | ☆ | 8 |
| Hu, 2016 | ★ | ★ | ★ | ★ | ★★ | ★ | ★ | ☆ | 8 |
| Manzi , 1995 | ★ | ★ | ★ | ★ | ★★ | ★ | ★ | ☆ | 8 |
| Nishimaki, 1999 | ★ | ★ | ★ | ★ | ★★ | ★ | ★ | ☆ | 8 |
| Pope, 2004 | ★ | ★ | ★ | ★ | ★☆ | ★ | ★ | ★ | 8 |
| Sayeeda, 2010 | ★ | ★ | ★ | ★ | ★☆ | ★ | ★ | ☆ | 7 |
| Strom, 1994 | ★ | ★ | ★ | ★ | ★★ | ★ | ★ | ★ | 9 |
| Zamora, 2020 | ★ | ★ | ★ | ★ | ★★ | ★ | ★ | ★ | 9 |

**(B) Study quality of cohort studies**

| **Author** | **Representativeness of the exposed cohort** | **Selection of the non exposed cohort** | **Ascertainment of exposure** | **Demonstration that outcome of interest was not present at start of study** | **Comparability of cohorts on the basis of the design or analysis** | **Assessment of outcome** | **Was follow-up long enough for outcomes to occur** | **Adequacy of follow up of cohorts** | **Total scores** |
| --- | --- | --- | --- | --- | --- | --- | --- | --- | --- |
| Borba, 2010 | ★ | ★ | ★ | ★ | ★★ | ★ | ★ | ★ | 9 |
| Chakravarty, 2013 | ★ | ★ | ★ | ★ | ★★ | ★ | ★ | ★ | 9 |
| Chen, 2011 | ★ | ★ | ★ | ★ | ★★ | ★ | ★ | ★ | 9 |
| Chen, 2014 | ★ | ★ | ★ | ★ | ★★ | ★ | ★ | ★ | 9 |
| Costa-Reis, 2013 | ★ | ☆ | ★ | ★ | ★★ | ★ | ★ | ★ | 8 |
| Da Silva, 2020 | ★ | ☆ | ★ | ★ | ★★ | ★ | ★ | ★ | 8 |
| Feldman, 2015 | ★ | ★ | ★ | ★ | ★★ | ★ | ★ | ★ | 9 |
| Ferreira, 2016 | ★ | ★ | ★ | ★ | ★★ | ★ | ★ | ★ | 9 |
| Frodlund, 2018 | ★ | ★ | ★ | ★ | ★★ | ★ | ★ | ★ | 9 |
| Garnier, 2018 | ★ | ★ | ★ | ★ | ★★ | ★ | ★ | ★ | 9 |
| Hata, 2011 | ★ | ★ | ★ | ★ | ★★ | ★ | ★ | ★ | 9 |
| Hsu, 2019 | ★ | ★ | ★ | ★ | ★★ | ★ | ★ | ★ | 9 |
| Kang, 2005 | ★ | ★ | ★ | ★ | ★★ | ★ | ★ | ★ | 9 |
| Kwan, 2022 | ★ | ★ | ★ | ★ | ★★ | ★ | ★ | ★ | 9 |
| Lee, 2006 | ★ | ★ | ★ | ★ | ★★ | ★ | ★ | ★ | 9 |
| Mok, 2023 | ★ | ★ | ★ | ★ | ★★ | ★ | ★ | ★ | 9 |
| Moutsopoulos, 1978 | ★ | ★ | ★ | ★ | ★★ | ★ | ★ | ★ | 9 |
| Nagasawa, 1990 | ★ | ☆ | ★ | ★ | ★★ | ★ | ★ | ☆ | 7 |
| Park, 2004 | ★ | ★ | ★ | ★ | ★☆ | ★ | ★ | ☆ | 7 |
| Rodziewicz, 2023 | ★ | ★ | ★ | ★ | ★★ | ★ | ★ | ★ | 9 |
| Ryu, 2021 | ★ | ★ | ★ | ★ | ★★ | ★ | ★ | ☆ | 8 |
| Teh, 2018 | ★ | ☆ | ★ | ★ | ★★ | ★ | ★ | ★ | 8 |
| Wu, 2011 | ★ | ★ | ★ | ★ | ★★ | ★ | ★ | ★ | 9 |
| Yang, 2018 | ★ | ★ | ★ | ★ | ★★ | ★ | ★ | ★ | 9 |
| Yu, 2022 | ★ | ★ | ★ | ★ | ★★ | ★ | ★ | ★ | 9 |
| Yun, 2016 | ★ | ★ | ★ | ★ | ★★ | ★ | ★ | ★ | 9 |

**(C) Study quality of cross-sectional studies**

| **Author** | **Define the source of information** | **List inclusion and exclusion criteria for exposed and unexposed subjects or refer to previous publications** | **Indicate time period used for identifying patients** | **Indicate whether or not subjects were consecutive if not population-based** | **Indicate if evaluators of subjective components of study were masked to other aspects of the status of the participants** | **Describe any assessments undertaken for quality assurance purposes** | **Explain any patient exclusions from analysis** | **Describe how confounding was assessed and/or controlled** | **If applicable, explain how missing data were handled in the analysis** | **Summarize patient response rates and completeness of data collection** | **Clarify what follow-up, if any, was expected and the percentage of patients for which incomplete data or follow-up was obtained** | **Total scores** |
| --- | --- | --- | --- | --- | --- | --- | --- | --- | --- | --- | --- | --- |
| Gormezano, 2015 | Yes | Yes | No | Yes | No | Yes | No | No | No | Yes | Unclear | 5 |
| Ishikawa, 1999 | Yes | Yes | No | No | Yes | Yes | No | No | No | Yes | Unclear | 5 |
| Kahl, 1994 | Yes | Yes | No | Yes | Yes | Yes | No | No | No | Yes | Unclear | 6 |
| Khalifa, 2007 | Yes | Yes | Yes | Yes | Yes | Yes | No | No | No | Yes | Unclear | 7 |
| Murray, 2016 | Yes | Yes | Yes | Yes | Unclear | Yes | Yes | Yes | Yes | Yes | Unclear | 9 |

**(D) Study quality of randomized controlled trials**

**Risk of bias graph**

**
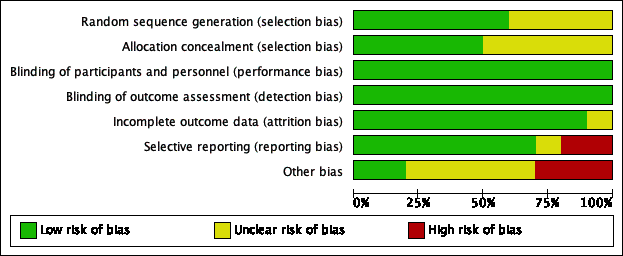
**

**Risk of bias summary**

**
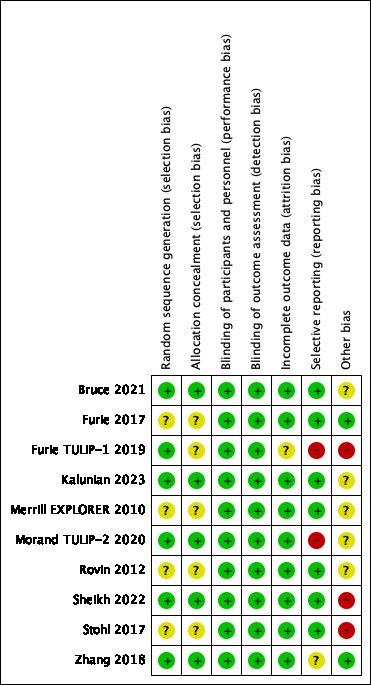
**

**Table S3.** Summary statistics of meta-regression of the prevalence of SLE-HZ.

| **Variables** | **Coef.** | **Standard error** | **t** | **P value** | **95% CI lower limit** | **95% CI upper limit** |
| --- | --- | --- | --- | --- | --- | --- |
| **Prevalence (%)** | | | | | | |
| **Region** |  | | | | | |
| Europe | 1 (Reference) | | | | | |
| Africa | -.0200866 | .1554361 | -0.13 | 0.898 | -.3395902 | .2994169 |
| Asia | .1028142 | .1120562 | 0.92 | 0.367 | -.1275206 | .333149 |
| North America | .0618487 | .1165803 | 0.53 | 0.600 | -.1777855 | .301483 |
| South America | .0584115 | .1194687 | 0.49 | 0.629 | -.18716 | .303983 |
| **Sample size** |  | | | | | |
| >1000 | 1 (Reference) | | | | | |
| <100 | .1823211 | .0415482 | 4.39 | **0.000** | .0972136 | .2674287 |
| 100-1000 | .0754978 | .0339323 | 2.22 | **0.034** | .0059906 | .145005 |
| **Publication year** |  | | | | | |
| 2014-2024  vs Before 2014 | .0584505 | .0382056 | 1.53 | 0.137 | -.0196887 | .1365897 |
| **SLE diagnostic criteria** |  | | | | | |
| Medical records | 1 (Reference) | | | | | |
| ACR-1982 | -.0210323 | .0700332 | -0.30 | 0.767 | -.1659071 | .1238425 |
| ACR-1997 | -.106917 | .0712212 | -1.50 | 0.147 | -.2542492 | .0404153 |
| Unclear (identify patient from ICD codes) | -.1038709 | .0731237 | -1.42 | 0.169 | -.2551388 | .047397 |
| **HZ diagnostic criteria** |  | | | | | |
| Unclear (identify patient from ICD codes) vs Classic clinical manifestations | -.0620456 | .0424528 | -1.46 | 0.155 | -.1491517 | .0250604 |
| **Incidence (per 1000 person-years)** | | | | | | |
| **Region** |  | | | | | |
| South America | 1 (Reference) | | | | | |
| Asia | 8.666443 | 9.123117 | 0.95 | 0.358 | -10.9007 | 28.23358 |
| Europe | 15.66889 | 23.79876 | 0.66 | 0.521 | -35.37438 | 66.71216 |
| North America | -3.951607 | 9.935613 | -0.40 | 0.697 | -25.26138 | 17.35816 |
| **Publication year** |  | | | | | |
| 2014-2024  vs Before 2014 | -2.268988 | 6.382669 | -0.36 | 0.727 | -15.79964 | 11.26167 |
| **SLE diagnostic criteria** |  | | | | | |
| ACR-1997 | 1 (Reference) | | | | | |
| ACR-1982 | 3.752628 | 10.09734 | 0.37 | 0.717 | -18.24759 | 25.75285 |
| Unclear (identify patient from ICD codes) | 4.010286 | 9.900248 | 0.41 | 0.693 | -17.5605 | 25.58107 |
| **HZ diagnostic criteria** |  | | | | | |
| Unclear (identify patient from ICD codes) vs Classic clinical manifestations | 3.491797 | 6.659244 | 0.52 | 0.607 | -10.62517 | 17.60876 |

Abbreviations: HZ=Herpes zoster; SLE=Systemic lupus erythematosus; ACR= American College of Rheumatology; ICD= International Classification of Diseases.

**
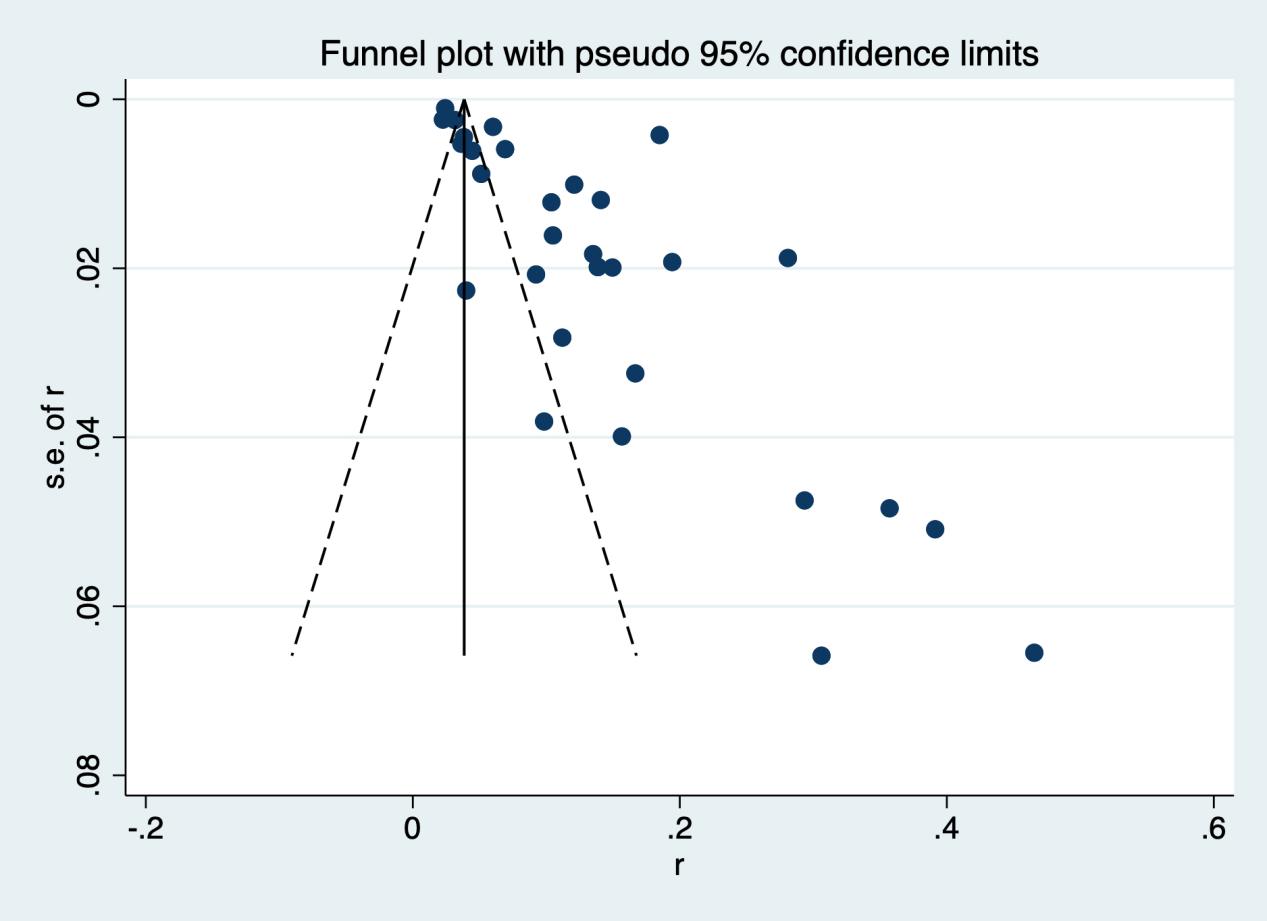
**

Figure S1. Funnel plots of publication bias for prevalence.


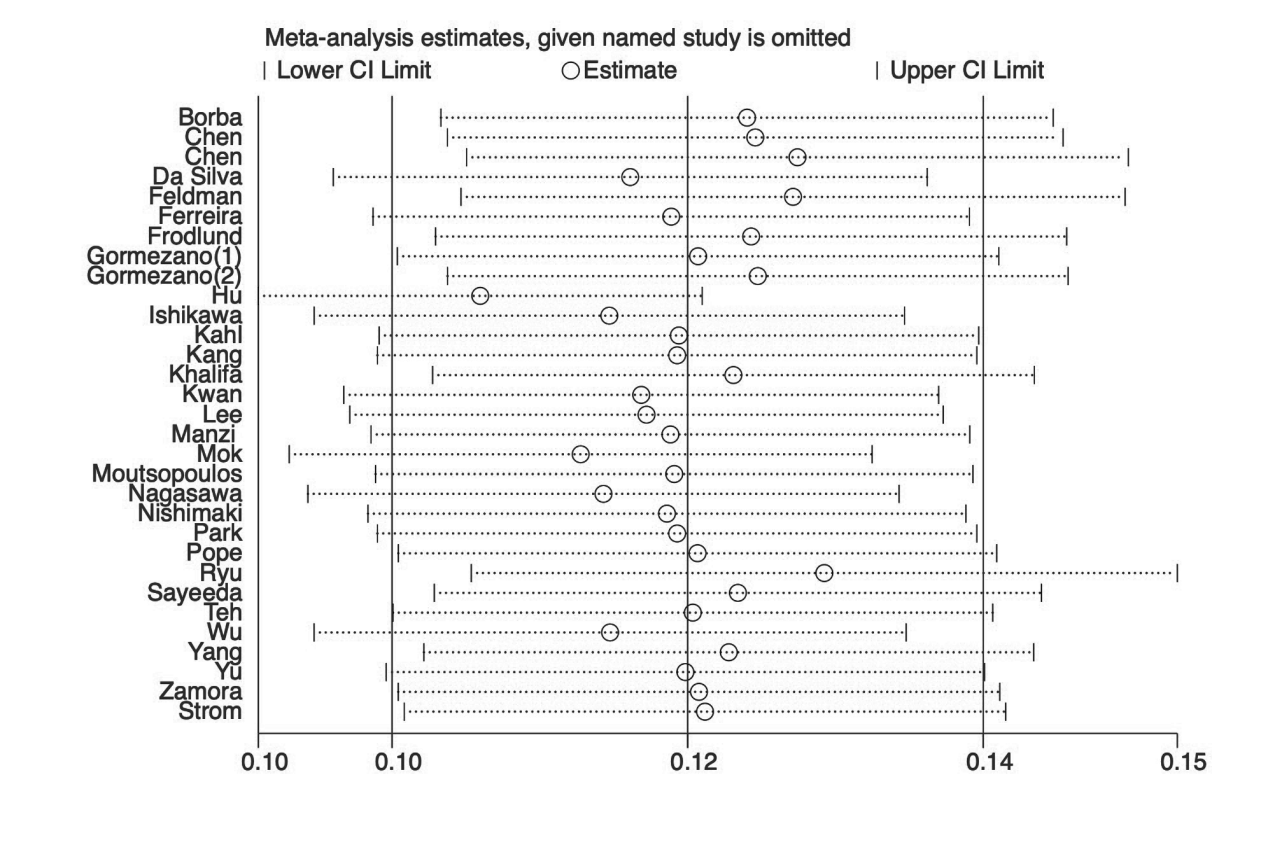


Figure S2. Sensitivity analysis of SLE-HZ prevalence. SLE, systemic lupus erythematosus; HZ, herpes zoster.

**
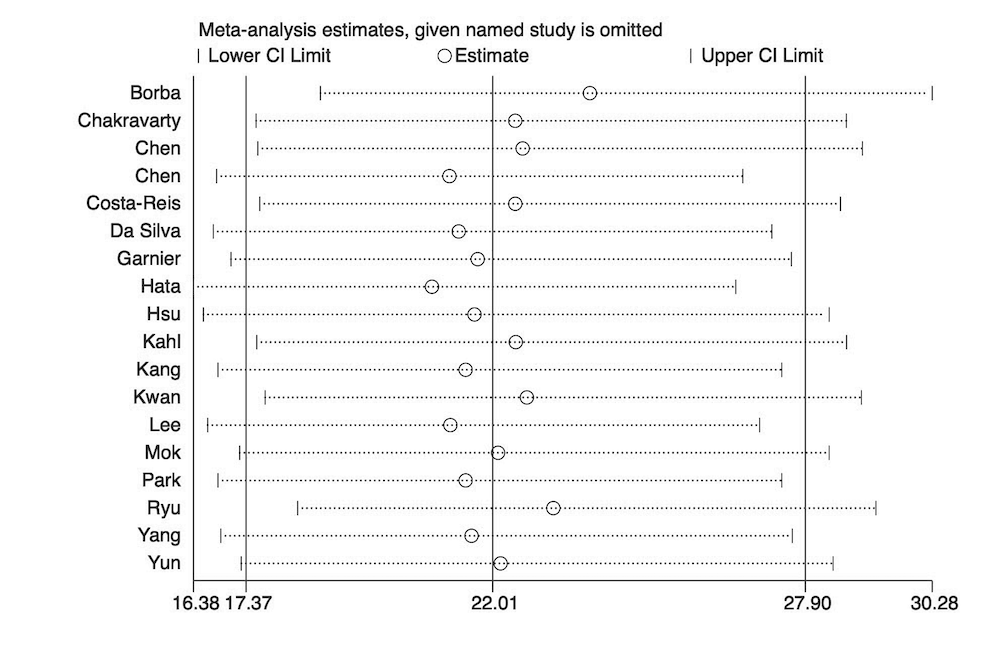
**

Figure S3. Sensitivity analysis of SLE-HZ incidence. SLE, systemic lupus erythematosus; HZ, herpes zoster.

**
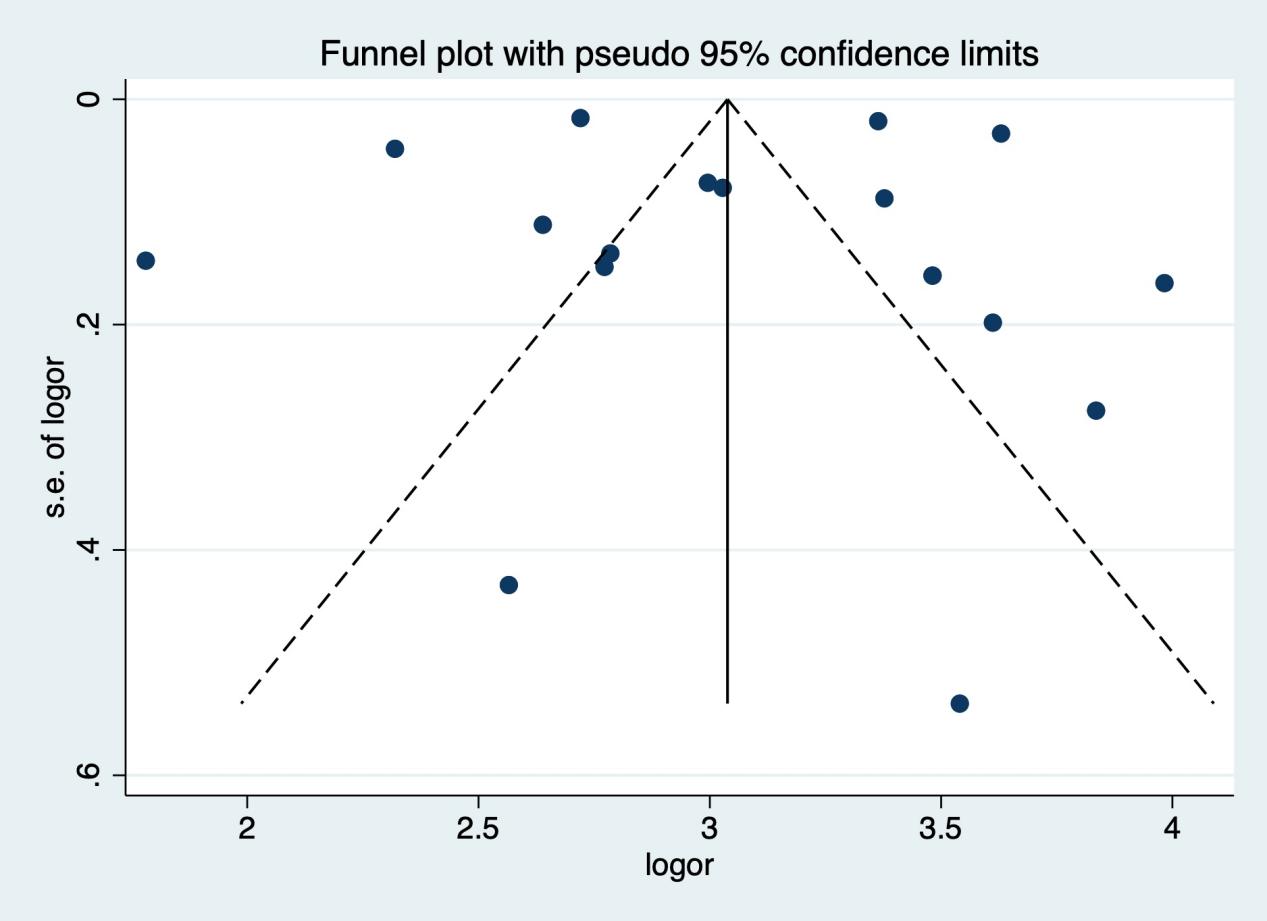
**

Figure S4. Funnel plots of publication bias for incidence.

**
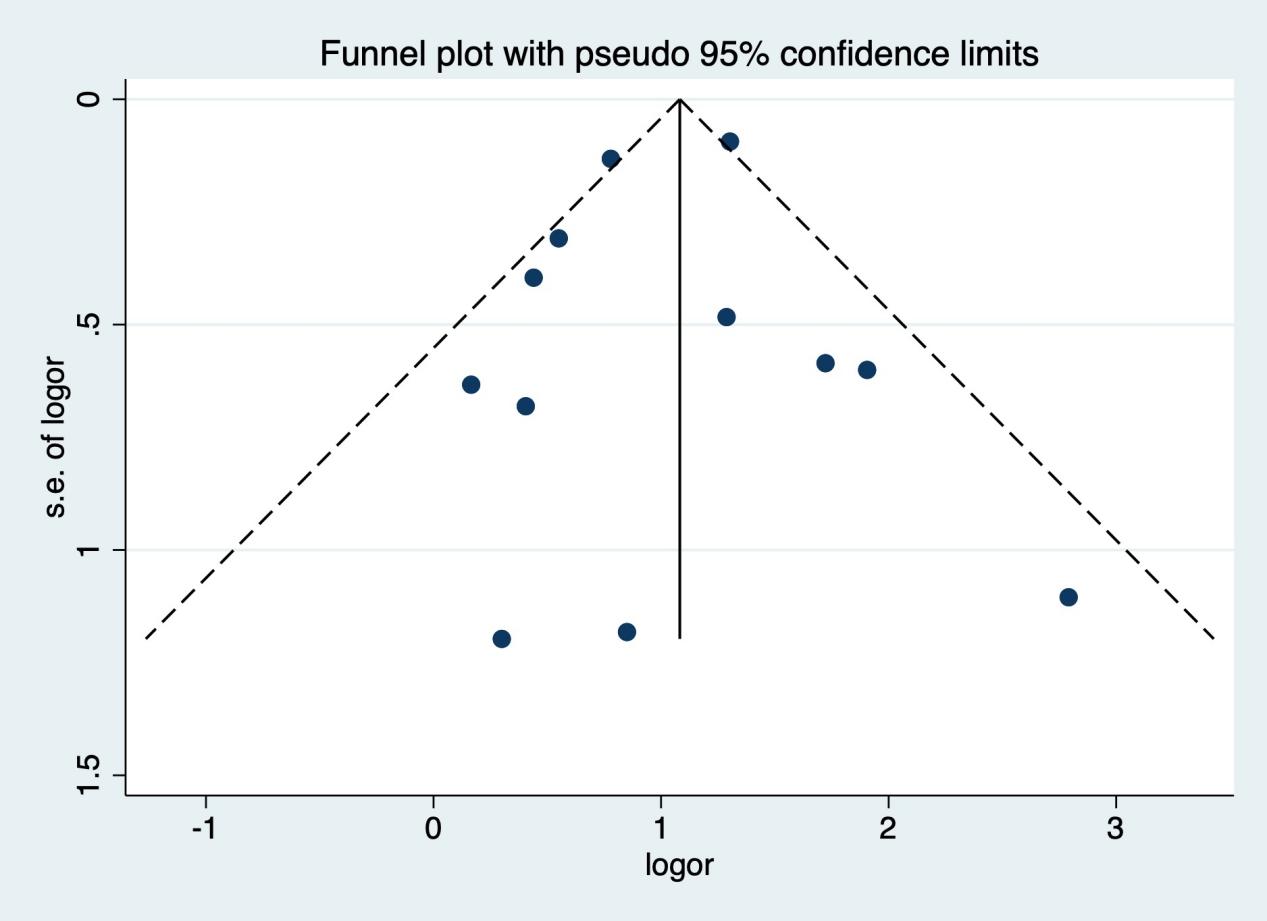
**

Figure S5. Funnel plots of publication bias for GCs usage. GCs, glucocorticoids.


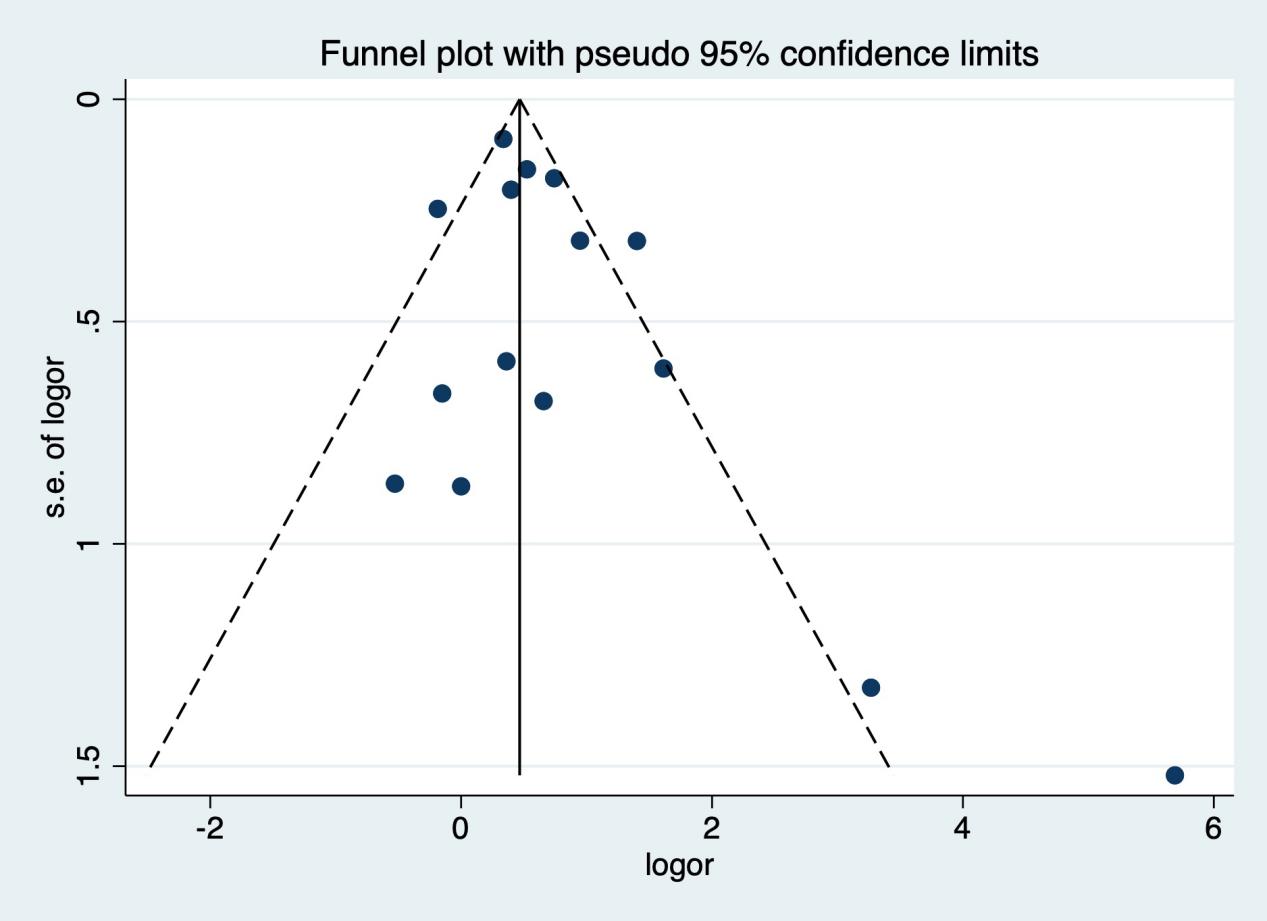


Figure S6. Funnel plots of publication bias for immunosuppressants usage.

**
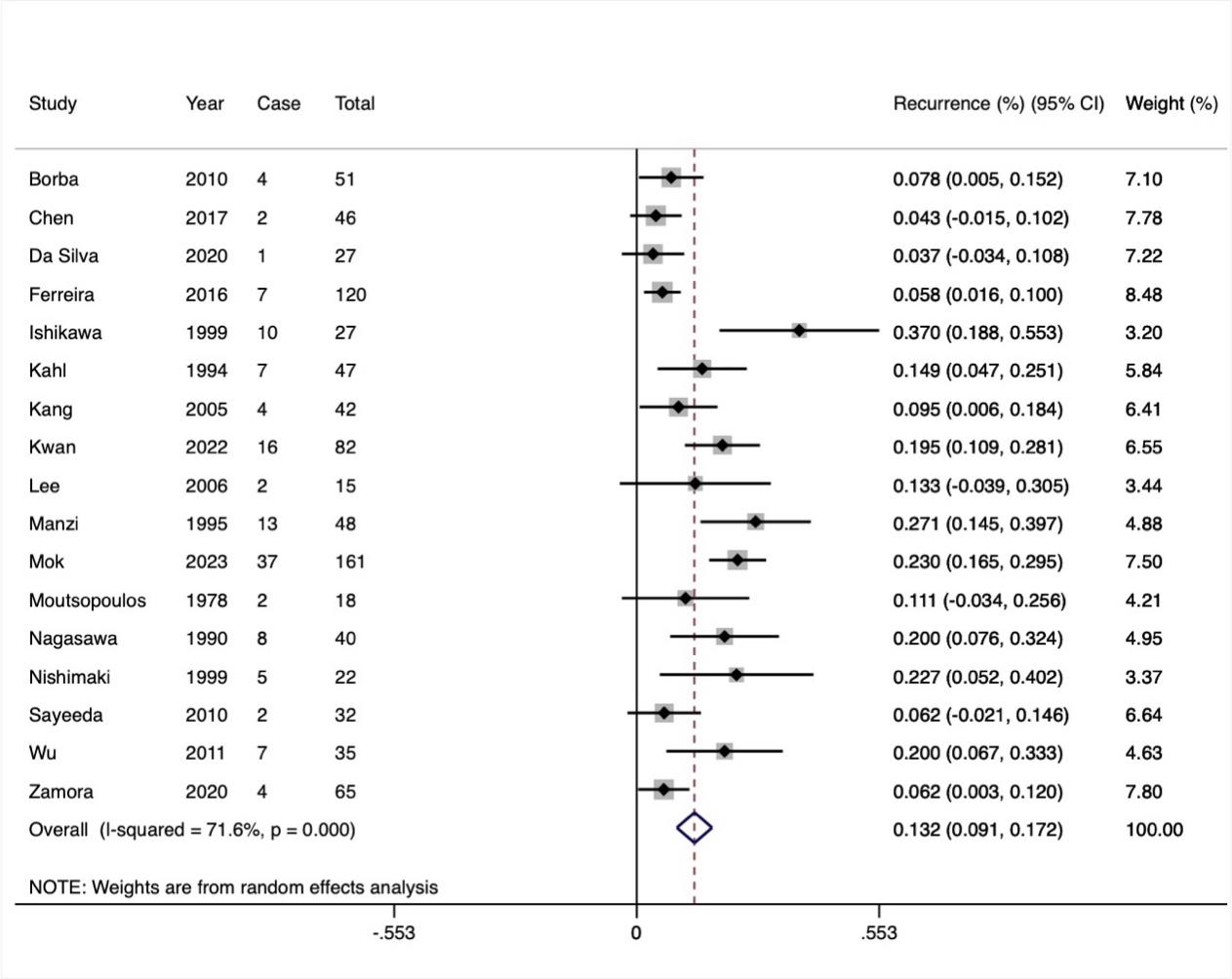
**

Figure S7. Forest plot of SLE-HZ recurrence. SLE, systemic lupus erythematosus; HZ, herpes zoster.

**
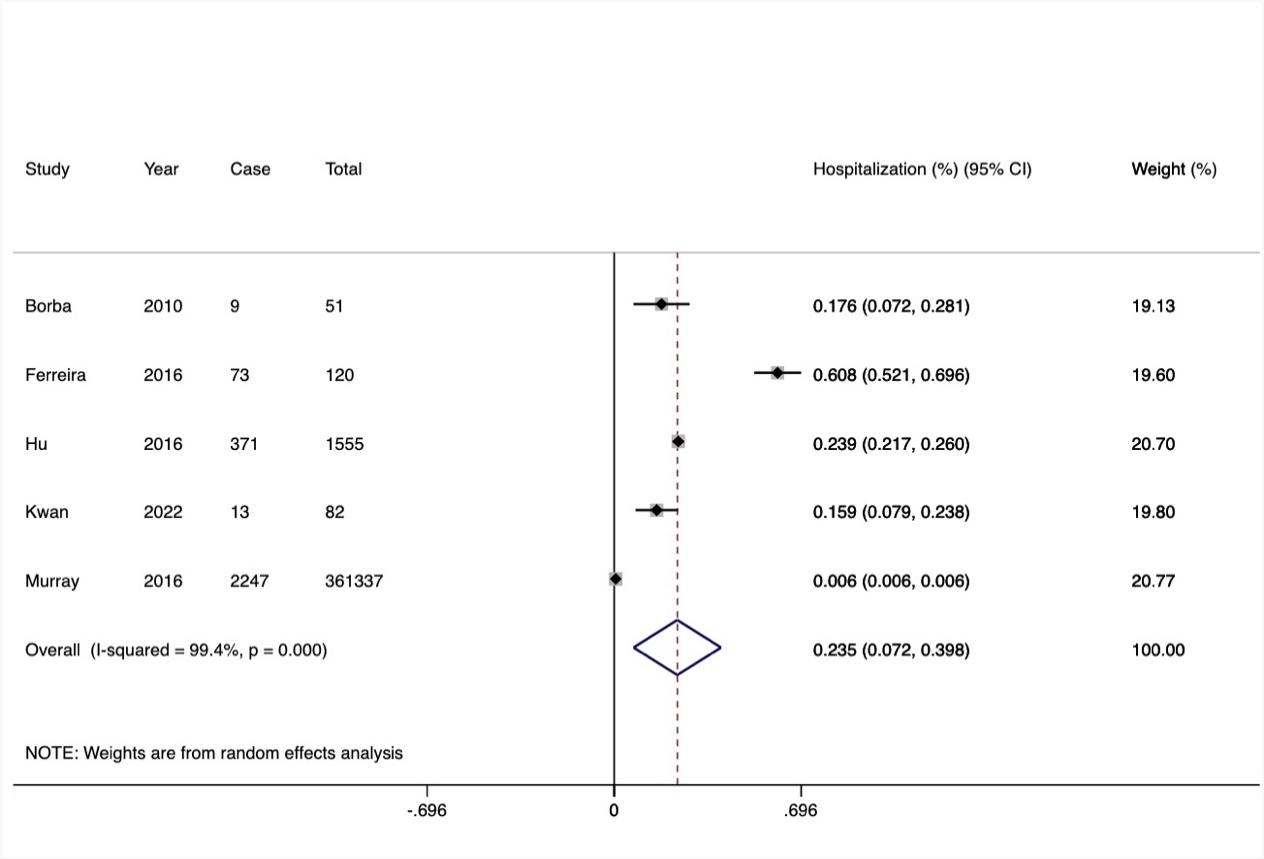
**Figure S8. Forest plot of the hospitalization of HZ in SLE patients. SLE, systemic lupus erythematosus; HZ, herpes zoster.


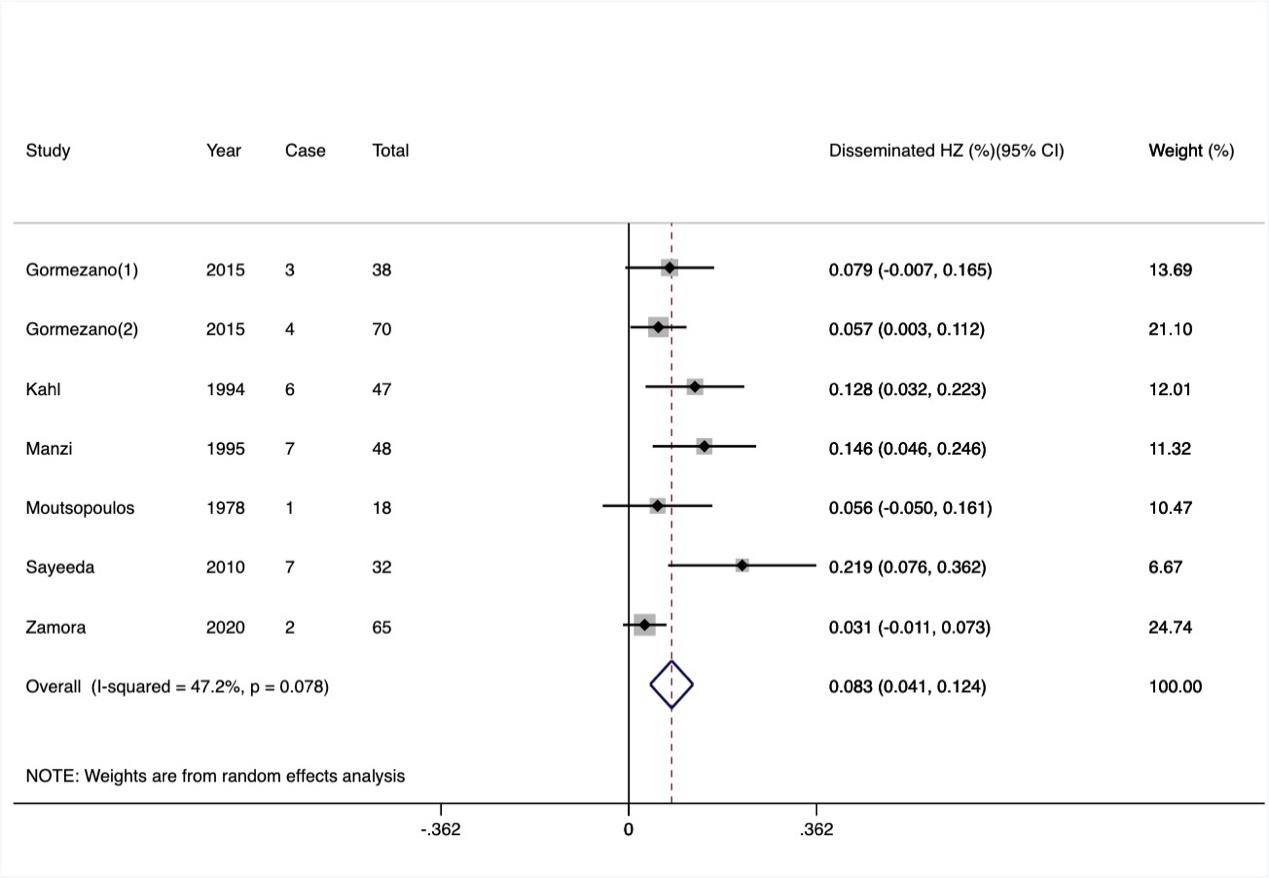


Figure S9. Forest plot of the disseminated HZ in SLE patients. SLE, systemic lupus erythematosus; HZ, herpes zoster.

**
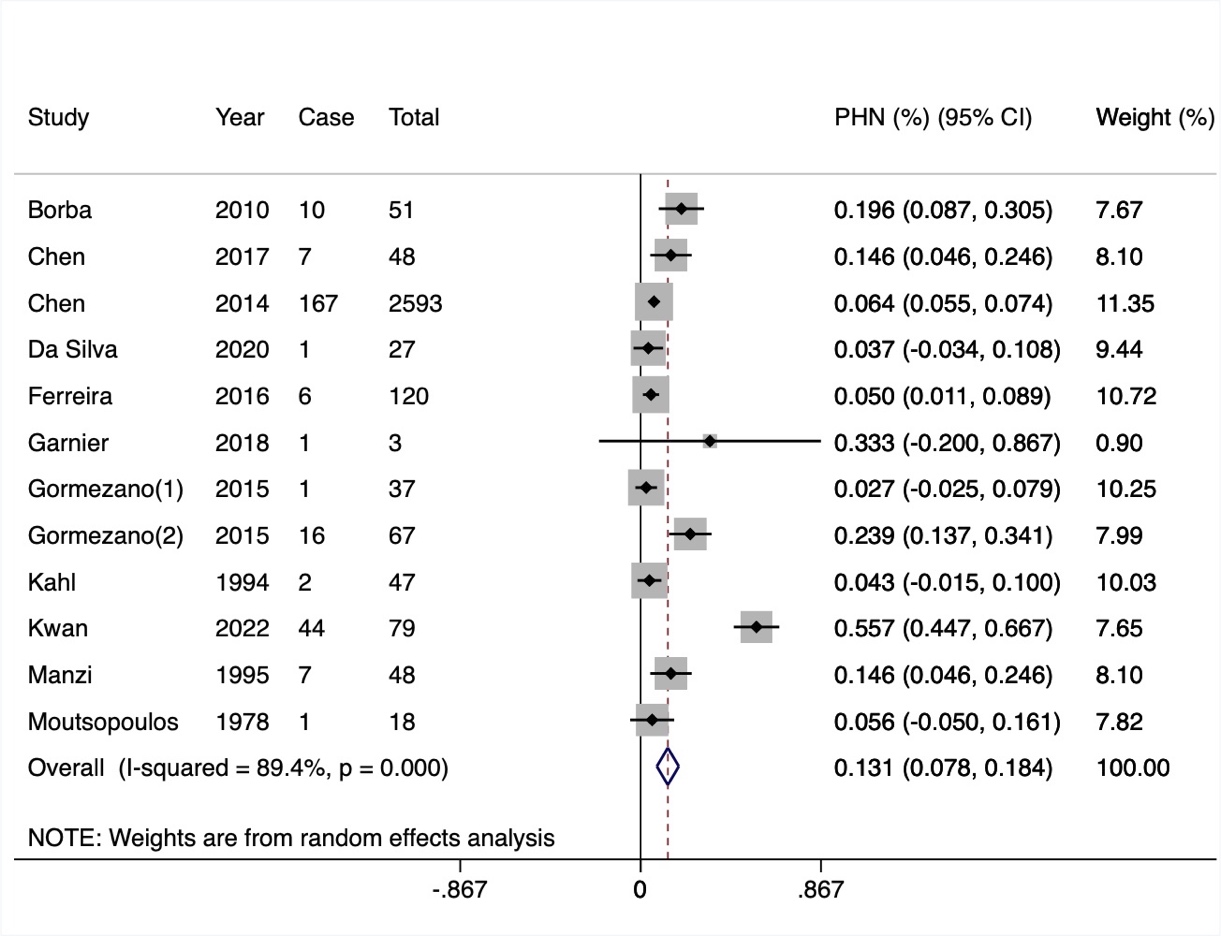
**

Figure S10. Forest plot of the PHN in SLE-HZ. PHN, post-herpetic neuralgia; SLE, systemic lupus erythematosus; HZ, herpes zoster.

**
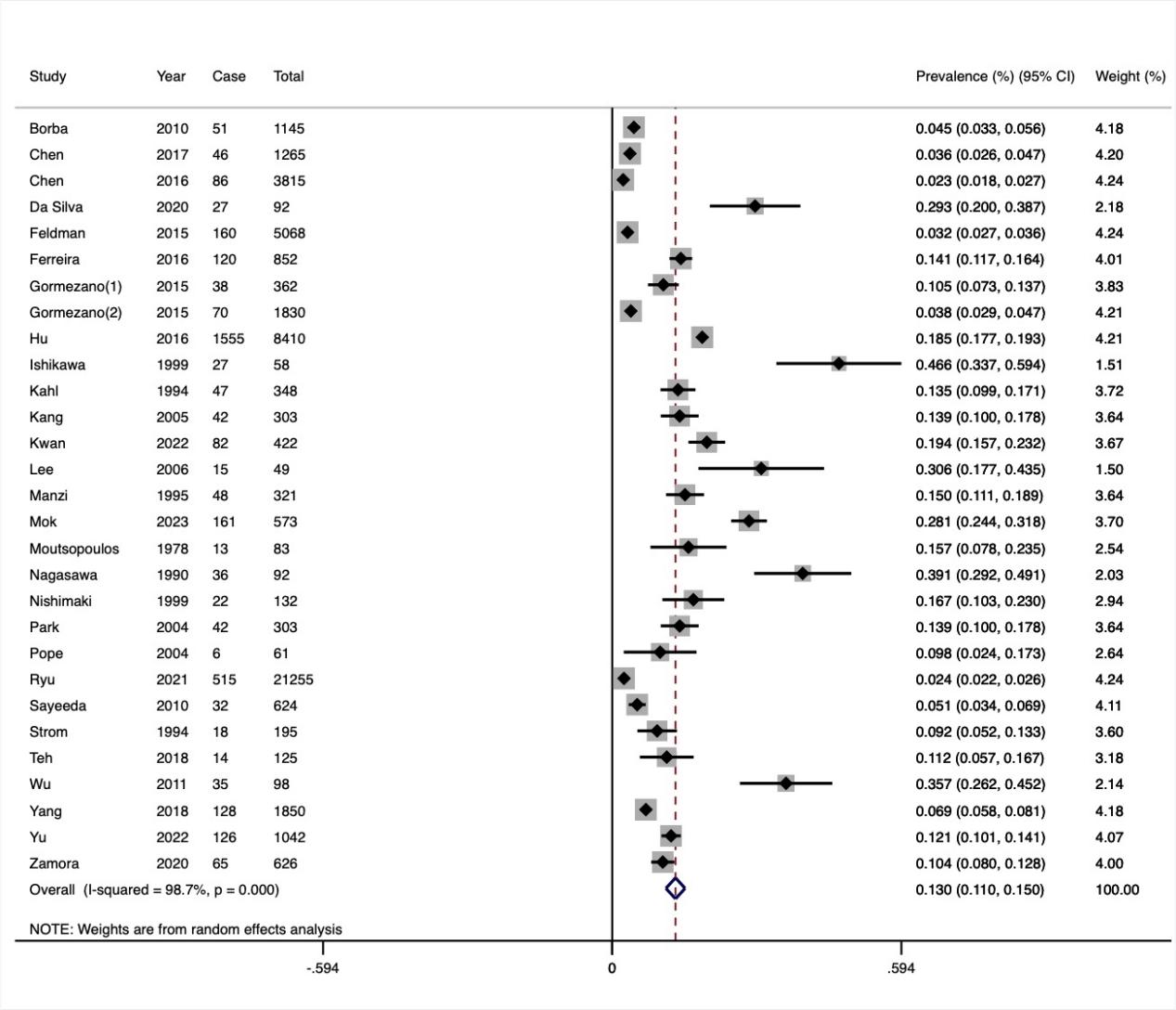
**

Figure S11. Forest plot of SLE-HZ prevalence after excluding two studies (one from Europe and one from Africa). SLE, systemic lupus erythematosus; HZ, herpes zoster.
